# Somatic mutations in *TBX3* promote hepatic clonal expansion by accelerating VLDL secretion

**DOI:** 10.1172/JCI191855

**Published:** 2025-07-10

**Authors:** Gregory Mannino, Gabriella Quinn, Min Zhu, Zixi Wang, Xun Wang, Boyuan Li, Meng-Hsiung Hsieh, Thomas Mathews, Lauren Zacharias, Wen Gu, Purva Gopal, Natalia Brzozowska, Peter Campbell, Matt Hoare, Glen Liszczak, Hao Zhu

**Affiliations:** 1 Children’s Research Institute, Department of Pediatrics and Internal Medicine, Center for Regenerative Science and Medicine, Simmons Comprehensive Cancer Center, Children’s Research Institute Mouse Genome Engineering Core;; 2 Department of Immunology; and; 3 Department of Pathology; University of Texas Southwestern Medical Center, Dallas, Texas, USA.; 4 Cancer Genome Project, Wellcome Sanger Institute, Hinxton, Cambridgeshire, United Kingdom.; 5 Quotient Therapeutics, Little Chesterford, Saffron Walden, United Kingdom.; 6 University of Cambridge Department of Medicine, Cambridge Biomedical Campus, Cambridge, United Kingdom.; 7 University of Cambridge Early Cancer Institute, Hutchison Research Centre, Cambridge Biomedical Campus, Cambridge, United Kingdom.; 8 Department of Biochemistry, University of Texas Southwestern Medical Center, Dallas, Texas, USA.

**Keywords:** Cell biology, Gastroenterology, Hepatitis

## Abstract

Somatic mutations that increase clone fitness or resist disease are positively selected, but the impact of these mutations on organismal health remains unclear. We previously showed that *Tbx3* deletion increases hepatocyte fitness within fatty livers. Here, we detected *TBX3* somatic mutations in patients with metabolic dysfunction–associated steatotic liver disease (MASLD). In mice, *Tbx3* deletion protected against, whereas *Tbx3* overexpression exacerbated, MASLD. *Tbx3* deletion reduced lipid overload by accelerating VLDL secretion. Choline-deficient diets, which block VLDL secretion, abrogated this protective effect. TBX3 transcriptionally suppressed the conventional secretory pathway and cholesterol biosynthesis. *Hdlbp* is a direct target of TBX3 that is responsible for the altered VLDL secretion. In contrast to wild-type *TBX3*, the *TBX3* I155S and A280S mutations found in patients failed to suppress VLDL secretion. In conclusion, *TBX3* mutant clones expand during MASLD through increased lipid disposal, demonstrating that clonal fitness can benefit the liver at the cost of hyperlipidemia.

## Abstract 2

*TBX3* somatic mutations were identified in the livers of patients with metabolic dysfunction-associated steatotic liver disease, and loss of *Tbx3* in mouse hepatocytes leads to clonal expansions. *Tbx3* deletion reduced lipid overload in the liver by accelerating VLDL secretion.

## Graphical abstract

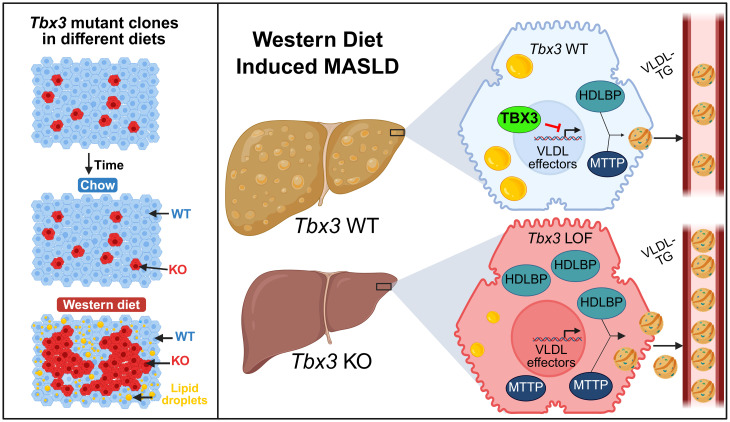

## Introduction

Somatic mosaicism results from the accumulation of mutations in subpopulations of cells within a tissue. Some somatic mutations are inconsequential, but others confer selective advantages that lead to clonal expansion within tissues. Recently, deep sequencing of normal tissues has revealed somatic mosaicism driven by clonal evolution in most healthy organs (1–3). While clonal evolution of hematopoietic stem cells has been extensively studied, somatic mosaicism within non-cancerous, solid tissues is just beginning to be investigated. In addition, it is now clear that the landscape of somatic mutations is determined not only by cell-autonomous influences of individual mutations, but also by environmental factors. Carcinogens such as those found in cigarette smoke increase somatic mosaicism within the lung and esophagus (4, 5). Chronic inflammation can affect the evolution of regenerating intestinal epithelial cells in ulcerative colitis patients (6–8). In the liver, chronic diseases exert specific selective pressures on expanding hepatocytes (9–11). Identifying mutations and understanding the mechanisms by which they induce clonal expansion are critical to understanding the relationship between distinct liver disease etiologies and hepatocyte expansion. To functionally explore somatic mutations in the liver, we recently developed an in vivo CRISPR screening platform called Method Of Somatic AAV-transposon In vivo Clonal Screening (MOSAICS) (11). This adeno-associated virus–based (AAV-based) platform induces somatic mosaicism in the liver through CRISPR/Cas9–mediated gene editing, which, when combined with different models of chronic liver disease, results in unique patterns of sgRNA enrichment and depletion. Using MOSAICS, we identified loss-of-function mutations that induce clonal expansion only in the presence of metabolic dysfunction–associated steatotic liver disease (MASLD), and one of the most highly enriched mutations was in the transcription factor *Tbx3* (11).

TBX3 is a transcription factor in the T-box family of DNA-binding proteins that is broadly expressed during embryogenesis in multiple tissues. *Tbx3*-null embryos die in utero (12–14), highlighting its importance during embryonic development. In humans, loss-of-function mutations in *TBX3* cause the developmental disorder ulnar mammary syndrome (15). Within the liver, *Tbx3* is expressed in zone 3 hepatocytes, downstream of canonical Wnt signaling (16). However, no link has been made between *TBX3* and chronic liver disease. Here, we show that *TBX3* mutations are enriched in clonally expanded hepatocytes in patients with MASLD. We demonstrate that loss of hepatic *Tbx3* ameliorates MASLD in Western diet–fed (WD-fed) mice, and elucidate the mechanisms driving clonal expansion and protection within MASLD. This offers an example of how somatic mutations can produce antagonistic relationships between cell, organ, and organismal fitness.

## Results

### Somatic mutations in TBX3 are observed in human livers.

**Figure 1:**
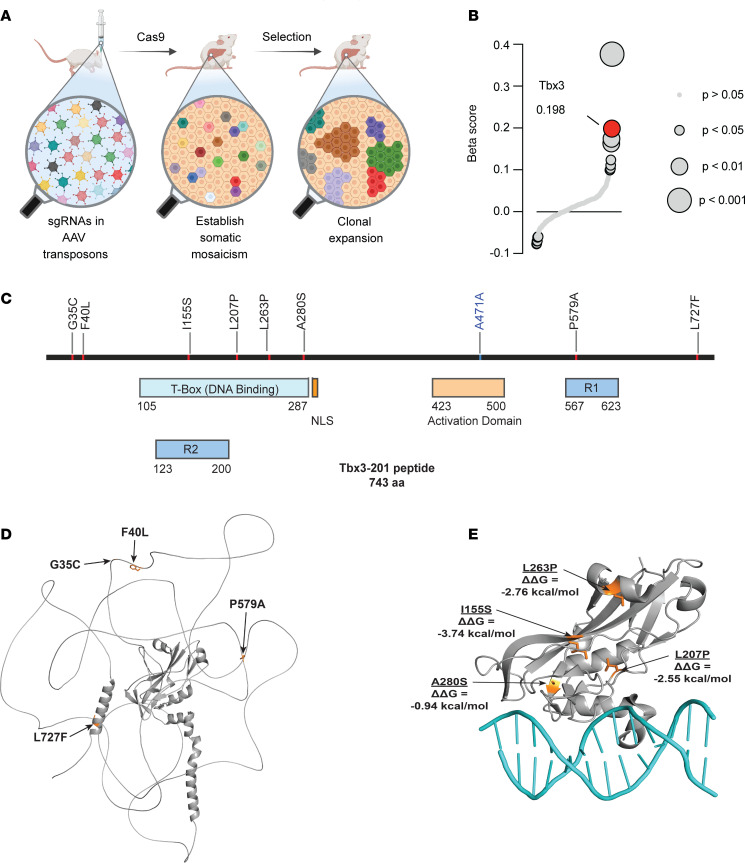
Somatic mutations identified in *TBX3* in liver tissues from MASLD patients. (**A**) Graphic of the MOSAICS screen workflow. (**B**) β-Score showing *sgTbx3* enrichment in WD- versus NC-fed mice after 6 months of selection. (**C**) Somatic mutations in *TBX3* identified from the livers of patients with MASLD. The mutation in blue represents a synonymous mutation. (**D**) AlphaFold-predicted TBX3 structure (gray) showing somatic mutations (orange) that do not fall within the DNA-binding domain. (**E**) X-ray crystal structure (PDB ID: 1H6F) of the TBX3 T-box (gray) bound to DNA (teal) showing somatic mutations (orange) with their respective ΔΔG values.

The MOSAICS platform permits the modeling of liver somatic mosaicism in different disease contexts, resulting in unique patterns of hepatocyte expansion and depletion (Figure 1A). Using MOSAICS, we previously observed that sgRNAs targeting the transcription factor *Tbx3* were among the most statistically significantly enriched under MASLD conditions (Figure 1B) (11), suggesting that *Tbx3* loss confers a selective advantage to hepatocyte clones during MASLD development.

We then asked whether somatic mutations in *TBX3* are present in humans with MASLD (Supplemental Table 1; supplemental material available online with this article; https://doi.org/10.1172/JCI191855DS1). Genomic sequencing of liver tissues from MASLD and alcohol-related liver disease patients identified somatic mutations in *TBX3* (10), several of which fell within the T-box DNA-binding domain (Figure 1C), indicating that *TBX3* mutations may also promote clonal expansion in human disease. To assess potential loss-of-function mechanisms, we mapped the mutations onto the AlphaFold-predicted structure of full-length TBX3. Mutations outside the DNA-binding domain had no predictable impact on TBX3 structure or function (Figure 1D). In contrast, mutations that occurred within the DNA-binding domain localized to key structural elements and were suggestive of loss-of-function mechanisms. To explore this further, we mapped the DNA-binding domain mutations onto the x-ray crystal structure of the TBX3 T-box bound to DNA (17). We then used DDMut to predict changes in Gibbs free energy (ΔΔG) between the WT and mutant proteins (18). The L207P, L263P, and I155S mutations were predicted to have moderate to severe destabilizing effects (ΔΔG ≤ –2.0 kcal/mol) (Figure 1E). Interestingly, the A280S mutation was predicted to only modestly destabilize TBX3 (ΔΔG = –0.94 kcal/mol), but is located near the DNA/protein interface (Figure 1E), suggesting that it may alter TBX3-DNA interactions. These findings suggested that several somatic *TBX3* mutations likely result in loss of functionality and could mirror the effects that were observed in mouse MASLD models.

### Tbx3 loss protects against MASLD development.

**Figure 2:**
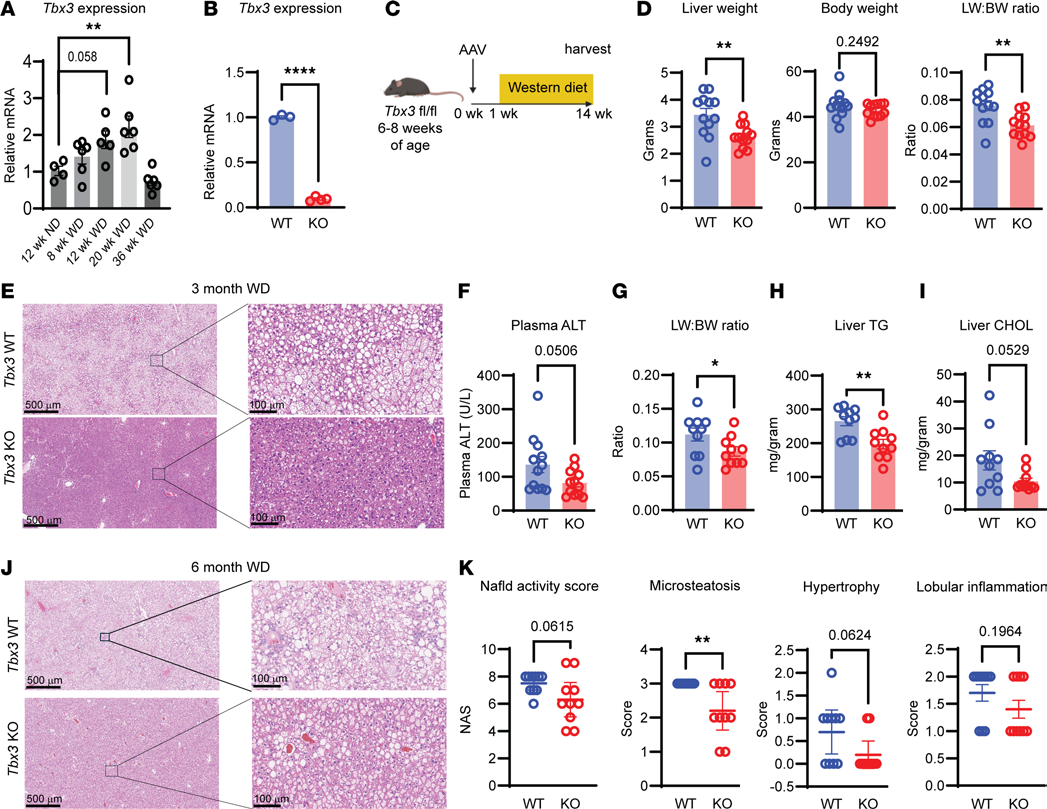
Liver-specific *Tbx3* deletion protects against MASLD. (**A**) *Tbx3* mRNA levels in WT mouse livers after NC or WD feeding for indicated durations. (**B**) *Tbx3* mRNA levels in *Tbx3^fl/fl^* livers 1 week after AAV-TBG-Cre injection. (**C**) Experimental schema for diet-induced MASLD. (**D**) Liver weight (left), body weight (middle), and liver/body weight ratio (right) of *Tbx3-*KO mice on WD for 3 months. (**E**) Representative H&E images from mice from **D**. (**F**) Plasma ALT levels from mice from **D**. (**G**) Liver/body weight ratio of *Tbx3*-KO mice on WD for 6 months. (**H**) Liver triglycerides from mice from **G**. (**I**) Liver cholesterol from mice from **G**. (**J**) Representative H&E images from mice from **G**. (**K**) NAS scores from mice from **G**. Significance of relative *Tbx3* mRNA in **A** was calculated using a 1-way ANOVA with Dunnett’s post hoc test. *P < 0.05; **P < 0.01; ****P < 0.0001. Scale bars: 500 μm, left panels; 100 μm, right panels.

To determine whether *Tbx3* levels physiologically change during MASLD progression, we performed quantitative PCR (qPCR) on livers from mice on a WD containing high fat, cholesterol, and sugar for up to 36 weeks. Compared with mice fed normal chow (NC) for 12 weeks, hepatic *Tbx3* expression was elevated in mice fed a WD for 8, 12, and 20 weeks, but decreased in mice fed a WD for 36 weeks (Figure 2A). To examine the role of *Tbx3* in MASLD development, we generated liver-specific *Tbx3*-knockout (*Tbx3*-KO) mice. Mice harboring *loxP* sites flanking exon 1 of *Tbx3* (14) were injected with AAV8-TBG-Cre, which leads to hepatocyte-specific *Cre* expression and loss of *Tbx3* mRNA expression in the liver (Figure 2B). To induce MASLD, we fed *Tbx3*-KO mice a WD for 3 months (Figure 2C). *Tbx3*-KO mice had decreased liver weights despite unchanged body weights, resulting in lower liver/body weight ratios (Figure 2D), suggesting reduced lipid accumulation. Compared with control mice, macrosteatosis and microsteatosis were attenuated in *Tbx3*-KO mice (Figure 2E) along with mRNAs involved in fibrosis (Supplemental Figure 1, A and B). We also observed reduced plasma alanine transaminase (ALT) in KO mice, indicative of reduced liver damage (Figure 2F). To ask whether the protection from MASLD could be sustained for longer periods, WT and KO mice were fed a WD for 6 months. Again, *Tbx3*-KO mice had lower liver/body weight ratios, liver triglycerides, and liver cholesterol (Figure 2, G–I). We then assessed the NAFLD activity score (NAS) (19) after 6 months of WD and found decreased NAS in KO versus WT mice (Figure 2, J and K). Despite the protection from MASLD, these mice had similar levels of liver fibrosis and plasma ALT (Supplemental Figure 1, C–E). Because apoptosis is known to play a role in MASLD progression, and TBX3 has been reported to regulate apoptosis, we checked *Tbx3-*KO livers for cleaved PARP, a common marker of apoptosis. However, we found no appreciable levels of cleaved PARP in either *Tbx3-*WT or -KO mice after 3 and 6 months of WD feeding (Supplemental Figure 1F). To determine whether this result is sex specific, we induced MASLD in *Tbx3-*KO female mice. While female mice develop less MASLD than male mice, *Tbx3-*KO females still showed reduced steatosis (Supplemental Figure 1, G and H).

We then asked whether *Tbx3* deletion would impact MASLD-induced cancer. We induced MASLD-driven tumorigenesis by feeding *Tbx3-*KO or -WT mice a WD for 48 weeks, allowing tumors to develop within the context of MASLD. After 48 weeks, *Tbx3-*KO mice again had decreased liver/body weight ratios (Supplemental Figure 2A). They also had decreased surface tumor numbers and sizes (Supplemental Figure 2, B and C). Together, these results suggest that liver-wide *Tbx3* loss is protective against WD-induced MASLD.

### Tbx3 overexpression exacerbates WD-induced MASLD development.

**Figure 3:**
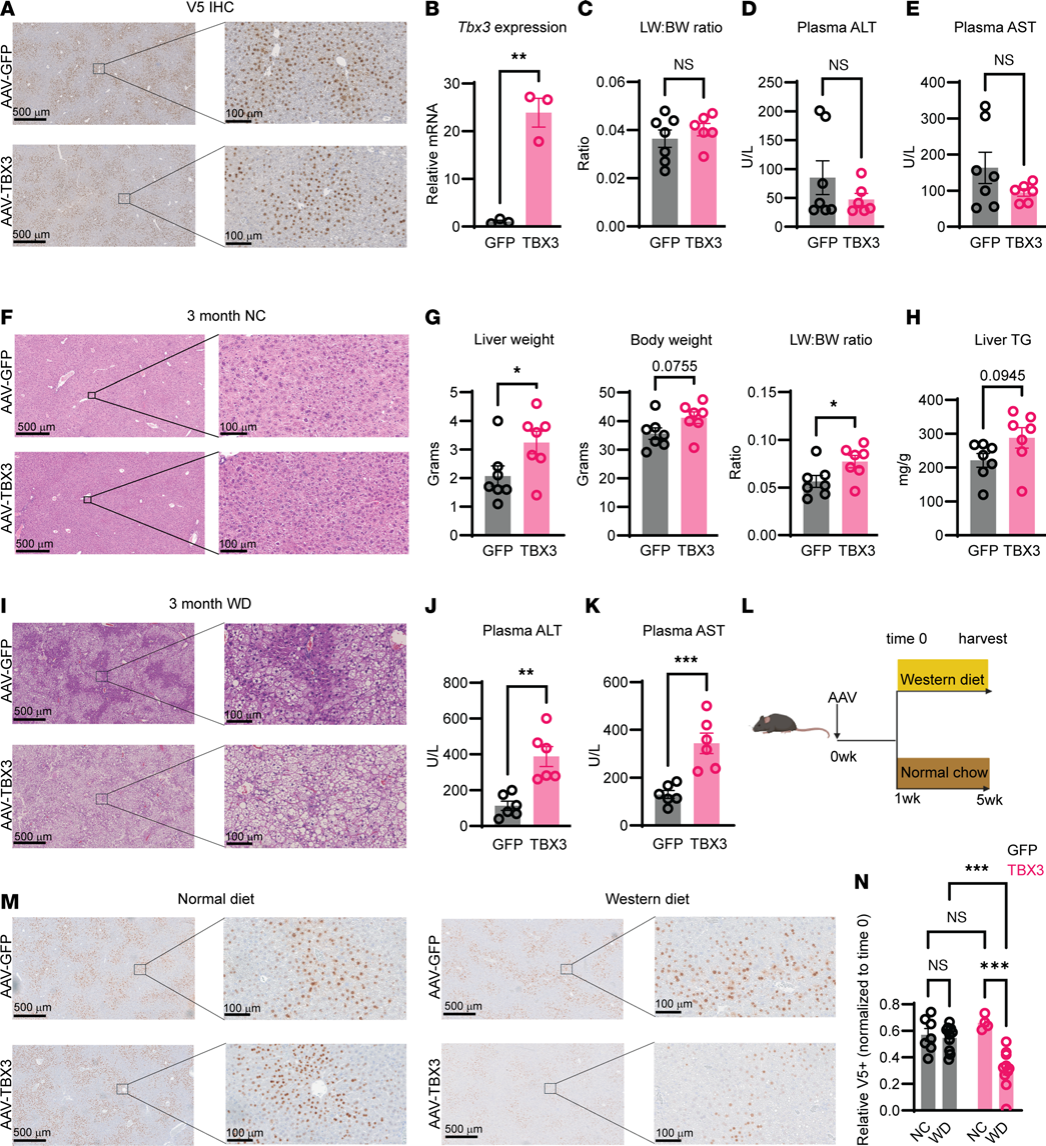
Overexpression of *Tbx3* exacerbates MASLD. (**A**) Representative V5 staining from WT mice injected with AAV8-TBG-V5-GFP or AAV8-TBG-V5-TBX3. (**B**) *Tbx3* mRNA levels from livers of mice overexpressing GFP or TBX3. (**C**)Liver/body weight ratio of *Tbx3*-overexpressing mice on NC for 3 months. (**D**) Plasma ALT from mice in **C**. (**E**) Plasma AST from mice in **C**. (**F**) Representative H&E images from mice in **C**. (**G**) Liver weight (left), body weight (middle), and liver/body weight ratio (right) of *Tbx3*-overexpressing mice on WD for 3 months. (**H**) Liver triglyceride measurements from mice in **G**. (**I**) Representative H&E images from mice in **G**. (**J**) Plasma ALT from mice in **G**. One outlier from each group was excluded. (**K**) Plasma AST from mice in **G**. One outlier from each group was excluded. (**L**) Experimental schema to test the fitness of *Tbx3-*expressing hepatocytes during WD feeding. (**M**) Representative V5 staining from WT mice injected with AAV8-TBG-V5-GFP or AAV8-TBG-V5-TBX3 after 4 weeks of NC or WD. (**N**) Quantification of relative abundance of V5-expressing cells from **H**. Significance of the V5^+^ quantification in **N** was calculated using a 2-way ANOVA with Tukey’s post hoc test. *P < 0.05; **P < 0.01; ***P < 0.001. Scale bars: 500 μm, left panels; 100 μm, right panels.

To determine whether increased *Tbx3* would be sufficient to promote MASLD, we generated an AAV8 overexpressing a V5-tagged *Tbx3* under the control of *TBG*, a hepatocyte-specific promoter (AAV8-TBG-V5-TBX3). One week after retro-orbital injection into WT mice, immunohistochemistry showed V5-positive staining and reverse transcription–qPCR confirmed increased *Tbx3* mRNA expression in the liver (Figure 3, A and B). After AAV delivery, we provided NC or WD for 3 months. On NC, *Tbx3* overexpression was insufficient to cause MASLD (Figure 3, C–F). On WD, *Tbx3* overexpression led to increases in liver weight, body weight, liver/body weight ratios, and liver triglycerides after 3 months (Figure 3, G and H). Histologically, overexpression led to increased steatosis and lipid droplet accumulation (Figure 3I). *Tbx3-*overexpressing mice also had increased plasma ALT and aspartate transaminase (AST), indicating increased liver damage (Figure 3, J and K). We observed increased *Tbx3* mRNA expression in livers after 3 months (Supplemental Figure 3, A and B), demonstrating that AAV-mediated overexpression was sustained for the entire feeding period. These results showed that *Tbx3* overexpression alone cannot drive MASLD, but can accelerate WD-induced MASLD.

Because *Tbx3*-KO hepatocytes are positively selected during MASLD (11), we asked whether forced expression of *Tbx3* would confer a selective disadvantage during MASLD. To test this, we overexpressed *Tbx3* in WT mice, followed by NC or WD feeding for 4 weeks (Figure 3L). After 4 weeks on an NC diet, there were similar levels of V5-positive cells in *GFP*- and *Tbx3*-overexpressing livers (Figure 3, M and N). However, after 4 weeks on WD, there were significantly fewer V5-positive cells in the *Tbx3*-overexpressing versus *GFP*-overexpressing control livers (Figure 3, M and N). One possibility is that *Tbx3* overexpression led to clonal demise in the context of WD-induced MASLD. Another possibility is that the selective pressure exerted by MASLD caused a subset of *Tbx3-*overexpressing hepatocytes to silence the AAV transgene in the context of WD feeding. Regardless of the mechanism, these results suggest that forced *Tbx3* expression in hepatocytes causes a selective disadvantage specifically in the context of MASLD, which is consistent with results showing that *Tbx3* deletion confers a selective advantage in the context of MASLD.

### Tbx3 loss does not improve fatty liver through altered insulin resistance.

**Figure 4:**
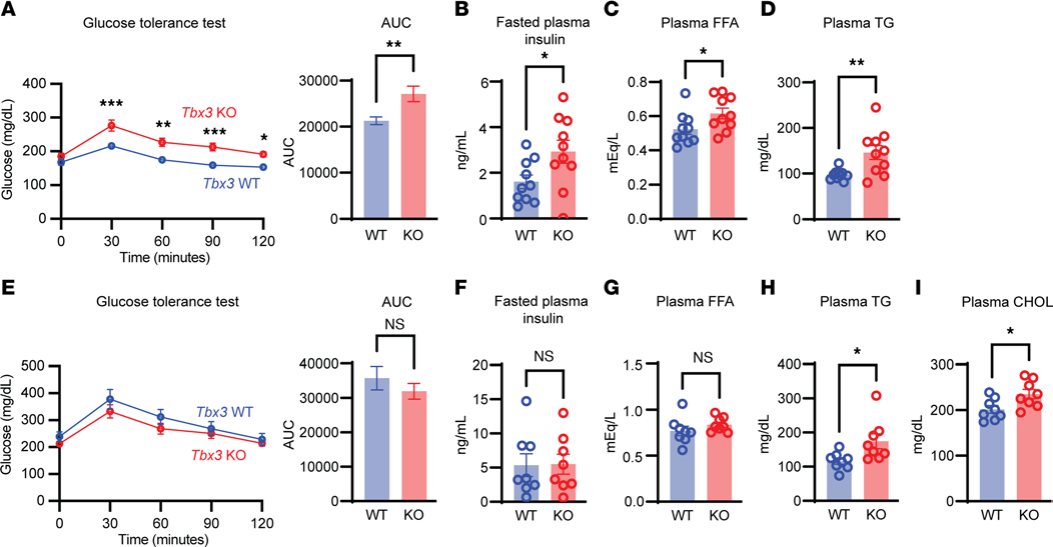
*Tbx3* loss exacerbates diet-induced metabolic syndrome. (**A**) Intraperitoneal glucose tolerance test from *Tbx3*-KO or -WT mice on WD for 6 months. *Tbx3* KO, *n* = 10; *Tbx3* WT, *n* = 10. (**B**) Fasting plasma insulin in mice from **A**. (**C**) Plasma free fatty acids (FFA) in mice from **A**. (**D**) Plasma triglycerides in mice from **A**. (**E**) Intraperitoneal glucose tolerance test in *Tbx3-*KO or -WT mice fed an NC diet for 6 months. *Tbx3* KO, *n* = 8; *Tbx3* WT, *n* = 8. (**F**) Fasted plasma insulin in mice from **E**. (**G**) Plasma FFAs in mice from **E**. (**H**) Plasma triglycerides in mice from **E**. (**I**) Plasma cholesterol in mice from **E**. Significance of the glucose tolerance test in **A** and **E** was calculated using a 2-way ANOVA with Šidák’s post hoc test. *P < 0.05; **P < 0.01; ***P < 0.001.

Because insulin resistance is correlated with MASLD progression (20), we asked whether *Tbx3*-KO mice were protected from MASLD through altered insulin sensitivity. We measured several metabolic parameters in *Tbx3*-KO mice, including glucose tolerance, fasting insulin levels, plasma non-esterified fatty acids, and plasma triglycerides. Surprisingly, *Tbx3*-KO mice exhibited increased glucose intolerance, fasting hyperinsulinemia, and increased plasma triglycerides and non-esterified fatty acids (Figure 4, A–D), all of which indicated increased insulin resistance. While selective insulin resistance seen in diabetic patients is a major driver of fatty liver disease, complete loss of hepatic insulin signaling is known to ameliorate MASLD (21), so we asked whether *Tbx3* might induce complete insulin resistance. To test this, we maintained *Tbx3*-KO mice on an NC diet for 6 months. Even on NC, *Tbx3*-KO mice trended toward lower liver weight and liver/body weight ratios, and had lower plasma ALT (Supplemental Figure 4). Under these conditions, *Tbx3*-KO mice had similar glucose tolerance, fasted insulin, and non-esterified fatty acids compared with WT mice (Figure 4, E–G). These mice still had elevated plasma triglycerides and plasma cholesterol (Figure 4, H and I). Overall, these results show that *Tbx3* loss alone is insufficient to drive insulin resistance, making complete insulin resistance unlikely to be responsible for MASLD protection.

### Tbx3 deletion transcriptionally upregulates genes involved in VLDL secretion.

**Figure 5:**
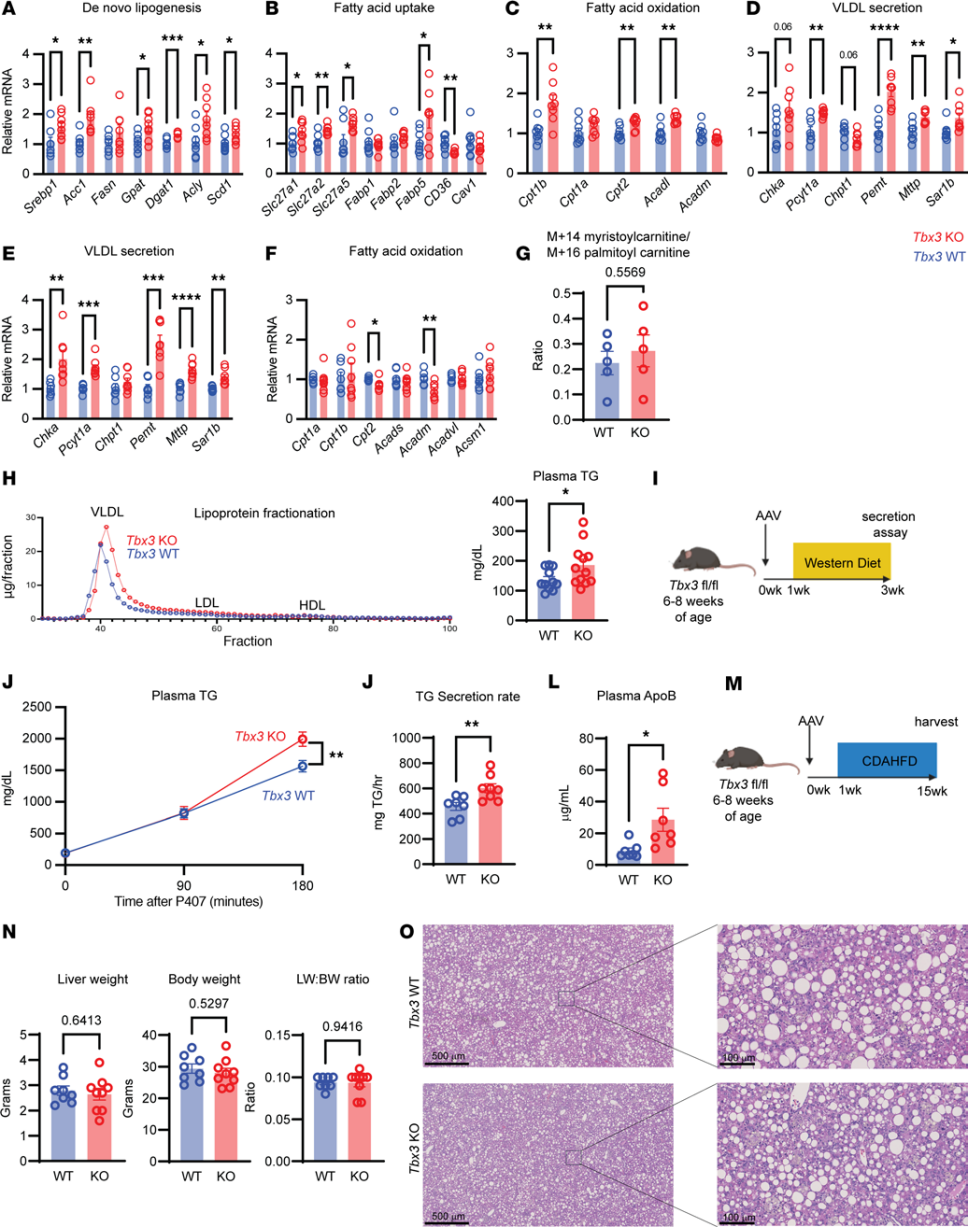
*Tbx3* deletion protects against MASLD by transcriptionally upregulating VLDL-TG particle secretion. (**A**) qPCR results for genes involved in de novo lipogenesis from *Tbx3-*KO or -WT mice fed a WD for 6 months. (**B**) qPCR for free fatty acid uptake genes in mice from **A**. (**C**) qPCR for β-oxidation genes in mice from **A**. (**D**) qPCR for PC biosynthesis and VLDL secretion genes in mice from **A**. (**E**) qPCR for PC biosynthesis and VLDL secretion genes in *Tbx3-*WT or -KO mice fed a WD for 4 weeks. (**F**) qPCR for β-oxidation genes in mice from **E**. (**G**) Ratio of M+14 myristoylcarnitine to M+16 palmitoylcarnitine in the liver of *Tbx3-*KO or -WT mice fed a WD for 2 weeks. (**H**) Lipoprotein fractionation (left) and total plasma triglyceride concentration (right) from *Tbx3-*KO mice fed a WD for 4 weeks. For lipoprotein fractionation, plasma was pooled from 4 mice per group. (**I**) Experimental setup for in vivo VLDL triglyceride secretion assay. (**J**) Plasma triglyceride levels over time from *Tbx3*-KO or -WT mice fed a WD for 2 weeks. *Tbx3* KO, *n* = 8; *Tbx3* WT, *n* = 7. (**K**) Quantification of the triglyceride secretion rate from mice from **K**. (**L**) Plasma concentration of total apolipoprotein B (ApoB) 3 hours after poloxamer injection in mice from **K**. (**M**) Experimental setup to induce MASLD with a CDA-HFD. (**N**) Liver weight (left), body weight (middle), and liver/body weight ratio (right) of *Tbx3*-KO or -WT mice fed a CDA-HFD for 14 weeks. (**O**) Representative H&E images in mice from **N**. 61. Significance of the difference in plasma triglycerides at 180 minutes after P407 injection in **J** was calculated using a 2-way ANOVA with Šidák’s post hoc test. *P < 0.05; **P < 0.01; ***P < 0.001; ****P < 0.0001. Scale bars: 500 μm, left panels; 100 μm, right panels.

We next investigated differences in major lipid metabolic pathways in the liver. The liver accumulates lipids primarily through de novo lipogenesis and free fatty acid uptake, while it disposes of lipids through fatty acid oxidation and very low-density lipoprotein (VLDL) particle secretion (22). After 6 months on a WD, *Tbx3*-KO mice had increased expression of de novo lipogenesis genes, including *Srebp1c*, *Acc1*, *Acly*, and *Scd1* (Figure 5A). Similarly, KO mice had elevated expression of genes involved in regulation of free fatty acid uptake, such as *Slc27a2*, *Slc27a5*, and *Fabp5* (Figure 5B). Upregulation of de novo lipogenesis and free fatty acid uptake are associated with insulin resistance, consistent with the metabolic syndrome in these mice, but are unlikely to mediate protection against MASLD. Next, we assessed regulators of fatty acid oxidation and VLDL secretion. Genes regulating fatty acid oxidation such as *Cpt1b*, *Cpt2*, and *Acadl* were upregulated (Figure 5C). In addition, critical genes for VLDL particle formation such as *Mttp* and *Sar1b* were upregulated (Figure 5D). Because phosphatidylcholine (PC) is the major phospholipid found in lipoproteins and known to be required for lipoprotein assembly (23), we also assessed components of the PC biosynthesis pathway. *Tbx3*-KO mice had elevated expression of PC biosynthesis genes including *Chka*, *Pcyt1a*, and *Pemt* (Figure 5D). These results suggested that *Tbx3* loss may protect from MASLD by increasing lipid disposal, either through increased fatty acid oxidation, VLDL secretion, or both.

Both fatty acid oxidation and VLDL secretion are impacted by the presence and severity of MASLD, so it is difficult to attribute transcriptional differences directly to loss of *Tbx3* versus differences caused by reduced MASLD. To disentangle these possibilities, we examined changes in these pathways early in MASLD progression, or after only 4 weeks of WD (Supplemental Figure 5, A and B). At this time point, we again found transcriptional upregulation of the PC biosynthesis and VLDL secretion genes, but no differences in fatty acid oxidation genes (Figure 5, E and F). These results suggested that upregulation of VLDL particle secretion occurs before fatty acid oxidation, making it more likely to be a direct effect of *Tbx3* loss.

Although we did not see a transcriptional upregulation in fatty acid oxidation early in disease, we wanted to ensure that *Tbx3* deletion was not increasing flux through β-oxidation to reduce MASLD. After 2 weeks of WD, we tested β-oxidation in *Tbx3-*KO mice using [^13^C]palmitate, a recently developed and validated in vivo tracing approach (24). We infused [^13^C]potassium palmitate into *Tbx3-*WT or -KO mice and performed metabolomics to calculate the ratio of M+14 myristoylcarnitine to M+16 palmitoylcarnitine to measure the rate of β-oxidation. We found that *Tbx3-*WT and -KO mice had similar rates of β-oxidation after 2 weeks on a WD (Figure 5G), suggesting that the *Tbx3* loss did not strongly perturb fatty acid oxidation.

While *Tbx3-*KO mice had increased expression of Slc27a2, Slc27a5, and Fabp5, we observed a decrease in the fatty acid transporter CD36 in *Tbx3-*KO mice after 6 months of WD feeding (Figure 5B). This prompted us to more directly investigate fatty acid uptake in *Tbx3-*KO mice. We performed lipidomics on *Tbx3-*KO mice infused with [^13^C]potassium palmitate after 2 weeks of WD feeding and measured fractional enrichment of M+16 palmitate in the liver and plasma of these mice. We observed no difference in M+16 palmitate enrichment in the plasma (Supplemental Figure 5C), confirming equivalent levels of tracer in the KO and WT mice. Similarly, we found no significant difference in M+16 palmitate enrichment in the liver (Supplemental Figure 5D), suggesting that *Tbx3* KO does not directly influence fatty acid uptake.

### Loss of Tbx3 accelerates VLDL particle secretion to protect against MASLD.

We further investigated the role of TBX3 in VLDL secretion. To understand the effect of *Tbx3* deletion on plasma lipids, we performed lipoprotein fractionation in *Tbx3*-KO mice. After 4 weeks on a WD, *Tbx3*-KO mice showed an increase in plasma triglycerides, specifically within the VLDL fractions (Figure 5H). To directly test the effect of *Tbx3* loss on VLDL particle secretion, we performed an in vivo VLDL-TG secretion assay using the detergent poloxamer-407 after 2 weeks of WD feeding (Figure 5I). KO mice had an increased rate of VLDL-TG secretion (Figure 5, J and K). Interestingly, we also found increased plasma apolipoprotein B (ApoB), suggesting that KO mice had increased numbers of VLDL-TG particles, rather than increased triglycerides per particle (Figure 5L). To test whether the increased VLDL secretion was the driving force behind the protection from MASLD, we used a choline-deficient, l-amino acid–defined high-fat diet (CDA-HFD) to induce MASLD (Figure 5M). This commonly used diet is deficient in both choline and methionine, which restricts PC biosynthesis, leading to lipoprotein retention, steatosis, and other features of MASLD (25). In contrast to mice fed a WD, *Tbx3*-WT and -KO mice had similar liver weight, body weight, and liver/body weight ratios (Figure 5N). Additionally, H&E showed equivalent levels of lipid accumulation in *Tbx3*-WT versus -KO livers (Figure 5O). *Tbx3-*KO mice also had similar levels of plasma ALT and AST, as well as similar levels of liver fibrosis (Supplemental Figure 6, A–D). Liver triglyceride measurements showed a small, but significant, decrease in *Tbx3-*KO mice (Supplemental Figure 6E), suggesting that the CDA-HFD is incapable of fully rescuing the anti-MASLD phenotype of *Tbx3* deletion. These results demonstrated that CDA-HFD impairs the central mechanism of *Tbx3* loss, namely accelerated VLDL particle secretion, thereby ablating the protective phenotypes associated with *Tbx3* deletion.

Because *Tbx3-*KO clones are positively selected as a result of increased VLDL-TG secretion, we asked whether altering VLDL secretion is a generalizable mechanism for MASLD-dependent clonal expansion. To see whether VLDL genes are associated with clonal fitness, we reanalyzed previous MOSAICS data (11). In WD-fed livers, *Mttp-*KO clones were significantly negatively selected, and *Tm6sf2-*KO clones trended toward negative selection (Supplemental Figure 6F). Notably, *Mttp* is required for the VLDL particle secretion, while *Tm6sf2-*KO clones can still secrete VLDL particles that are triglyceride poor. These results suggest that secreting lipids, rather than storing them, is a general mechanism of hepatocyte protection that is exemplified by *Tbx3-*KO clones.

### TBX3 suppresses VLDL secretion through regulating HDLBP.

**Figure 6:**
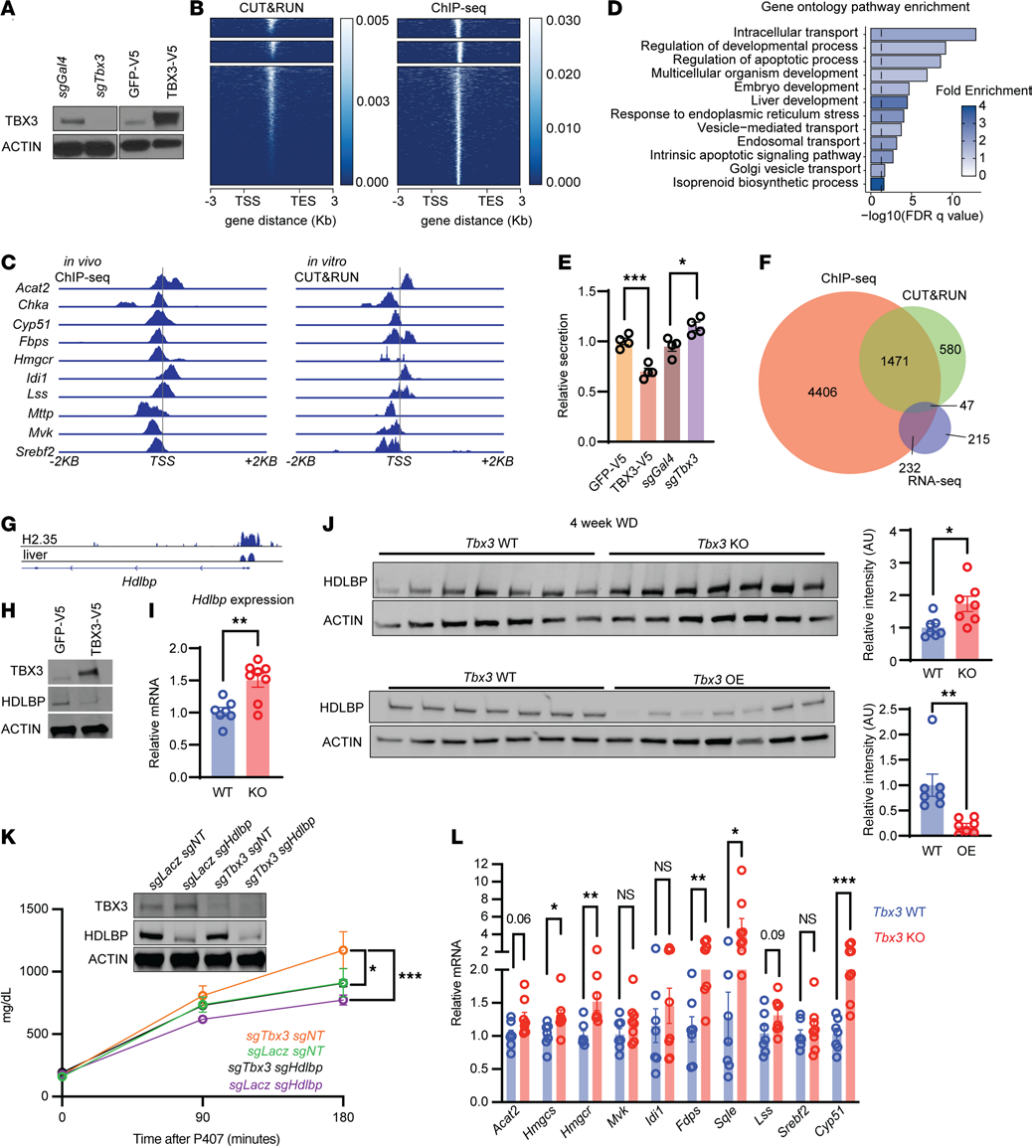
TBX3 regulates VLDL secretion through regulating cholesterol homeostasis. (**A**) Western blots showing *Tbx3* KO and overexpression in H2.35 cells. (**B**) Heatmaps from in vitro CUT&RUN (left) and in vivo ChIP-Seq for TBX3 binding loci. (**C**) Genomic tracks of VLDL secretion and cholesterol biosynthesis genes showing TBX3 binding in vitro and in vivo. (**D**) Gene Ontology pathway enrichment analysis from overlapping in vitro and in vivo TBX3 binding sites. (**E**) Relative secretion of Gaussia luciferase from *Tbx3-*KO and overexpression H2.35 cell lines. (**F**) Venn diagram showing the overlap of genes that are transcriptionally upregulated during MASLD in *Tbx3-*KO livers and have a TBX3 binding site in vitro and in vivo. (**G**) *Hdlbp* genomic tracks showing TBX3 binding in vitro and in vivo. (**H**) Western blot showing HDLBP expression in *Tbx3-*overexpressing H2.35 cells. (**I**) qPCR of *Hdlbp* mRNA levels in livers from *Tbx3-*KO mice fed a WD for 4 weeks. (**J**) Western blot showing HDLBP protein levels in livers from *Tbx3-*KO mice (top) or *Tbx3-*overexpressing mice (bottom) fed a WD for 4 weeks. (**K**) Western blot showing *Tbx3* and *Hdlbp* double knockout (DKO) in vivo and triglyceride secretion assay from *Tbx3/Hdlbp* DKO mice fed a WD for 2 weeks (*sgLacZ*/*sgNT*, *n* = 7; *sgLacZ*/*sgHdlbp*, *n* = 7; *sgTbx3*/*sgNT*, *n* = 7; *sgTbx3*/*sgHdlbp*, *n* = 8). (**L**) qPCR of cholesterol biosynthesis genes from *Tbx3-*KO or -WT mice fed a WD for 4 weeks. Significance of the difference in plasma triglycerides among all groups at 180 minutes after P407 injection in **K** was calculated using a 2-way ANOVA with Tukey’s post hoc test. *P < 0.05; **P < 0.01; ***P < 0.001.

To determine the transcriptional targets of TBX3 in the context of MASLD, we generated *Tbx3*-KO and V5-tagged *Tbx3* overexpression hepatocyte cell lines (Figure 6A). To identify genome-wide TBX3 binding sites, we performed CUT&RUN on *Tbx3*-overexpressing cells in tandem with Ty1-TBX3 ChIP-Seq on *Ty1-TBX3*–overexpressing mouse livers (Figure 6B). When we intersected these in vitro and in vivo data, we identified 1,518 genes containing a TBX3 binding site within 10 kb of their transcriptional start sites. Among these loci were *Mttp* and *Chka*, canonical regulators of VLDL secretion and PC biosynthesis, respectively. This suggested that TBX3 directly regulates some VLDL secretory components (Figure 6C). We then subjected the genes associated with all overlapping hits to Gene Ontology pathway enrichment analysis (Figure 6D). This identified several pathways related to developmental processes and apoptosis, known functions of TBX3 (12).

This approach also identified enrichments in intracellular transport and ER/Golgi vesicle–mediated transport pathways. Because loss of *Tbx3* accelerates the secretion of VLDL particles, and several secretory pathway genes were also enriched with TBX3 binding sites, we asked whether TBX3 was specifically regulating VLDL-TG, or secretion more generally. We tested secretory function using a Gaussia luciferase assay. Gaussia luciferase is a 20 kDa protein isolated from the marine copepod *Gaussia princeps*, and is normally secreted through the conventional secretory pathway (26). After *Gaussia* luciferase transfection into *Tbx3*-KO and -overexpressing H2.35 hepatocyte cell lines, along with their respective controls, we measured luciferase activity in cell culture medium. While *Tbx3* overexpression suppressed secretion, *Tbx3* deletion accelerated it (Figure 6E). To test secretion using other knockdown approaches in human cells, we cotransfected HEK293T cells with Gaussia luciferase and 3 independent siRNAs targeting *TBX3*. Forty-eight hours after transfection, all 3 siRNAs led to increased Gaussia luciferase secretion (Supplemental Figure 6G). This showed that TBX3 is a transcriptional regulator of secretory pathways that include VLDL.

To identify targets of TBX3 that may be mediating the enhanced secretion of VLDL in the MASLD context, we overlapped TBX3 binding sites with RNA sequencing data generated in WD-fed mice with liver-specific *Tbx3* knockout, generated with Cas9 and AAV-U6-sgTbx3 (11). Because TBX3 has transcriptional suppressor functions, we overlapped our binding sites with mRNAs that were upregulated (Figure 6F). We identified 47 genes bound by TBX3 and upregulated in KO livers during MASLD development. Among these gene products, HDL binding protein (HDLBP) is an RNA-binding protein that enhances the translation and secretion of bound mRNAs destined for ER-Golgi secretion (27). More specifically, HDLBP is known to bind and enhance the translation of *ApoB100* mRNA, thereby increasing VLDL-TG secretion (28). Through this mechanism, HDLBP is a critical regulator of cholesterol homeostasis and prevents excess intracellular cholesterol. In vitro and in vivo, TBX3 binds to sites within 250 bp of *Hdlbp*’s promoter (Figure 6G). In H2.35 hepatocytes, *Tbx3* overexpression suppressed HDLBP levels (Figure 6H). In vivo, *Tbx3*-KO livers showed elevated *Hdlbp* mRNA and protein after 4 weeks of WD (Figure 6, I and J), whereas *Tbx3*-overexpressing livers showed reduced levels after 4 weeks of WD (Figure 6J). Altogether, TBX3 directly suppresses HDLBP in vitro and in vivo. To test whether *Hdlbp* upregulation was causing enhanced VLDL secretion as a result of *Tbx3* deletion, we used inducible Cas9 mice and AAV-U6-sgRNAs to generate *Tbx3*/*Hdlbp* double-knockout (DKO) livers (Figure 6K). This showed that loss of *Hdlbp* abrogated the increases in VLDL-TG secretion caused by loss of *Tbx3* (Figure 6K). These results demonstrated that *Hdlbp* is a key downstream target of TBX3 that mediates the enhanced secretion of VLDL particles.

We asked whether TBX3 might regulate cholesterol homeostasis more generally beyond HDLBP. Interestingly, isoprenoid biosynthesis genes were also enriched in TBX3 binding sites (Figure 6D). Several genes that regulate cholesterol biosynthesis and homeostasis contain TBX3 binding sites within their promoters (Figure 6C). Moreover, many cholesterol synthesis genes were upregulated in *Tbx3*-KO livers after 4 weeks on a WD (Figure 6L). Even in the absence of WD feeding, loss of *Tbx3* led to transcriptional upregulation of several cholesterol biosynthesis genes (Supplemental Figure 6H), further showing that TBX3 directly suppresses the cholesterol biosynthetic pathway. Taken together, our results suggest that TBX3 transcriptionally regulates cholesterol synthesis and export to control VLDL particle secretion.

### Human TBX3 point mutations result in loss of functionality.

**Figure 7:**
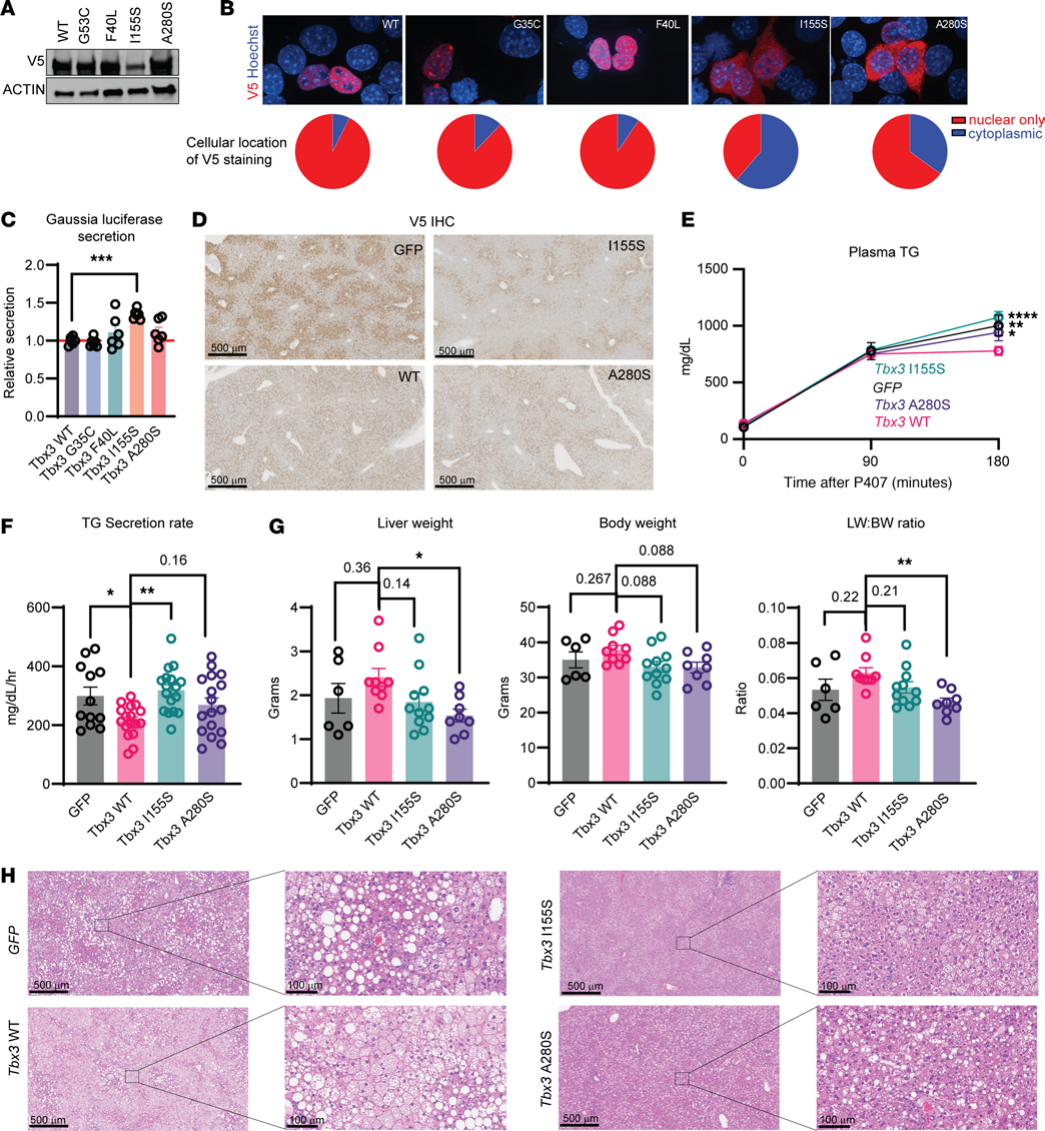
Human *TBX3* point mutations are loss-of-function. (**A**) Western blot for V5 from WT H2.35 cells transfected with WT or mutant TBX3. (**B**) Immunofluorescence for V5 from WT H2.35 cells transfected with WT or mutant *TBX3*. (**C**) Relative secretion of Gaussia luciferase from WT H2.35 24 hours after cotransfection with a Gaussia luciferase expression plasmid along with WT or mutant *Tbx3* expression plasmid. The significance in **C** was calculated using a 1-way ANOVA comparing each group to the *Tbx3* WT group and corrected with Dunnett’s post hoc test. (**D**) Representative immunohistochemistry images of V5^+^ cells in livers of WT C57BL/6 mice 1 week after AAV injection expressing *GFP*, WT *Tbx3*, or *Tbx3* harboring the I155S or A280S mutation. Scale bars: 500 μm. (**E**) Triglyceride secretion assay after 2 weeks of WD feeding from mice from **D** (*GFP*, *n* = 12; *Tbx3* WT, *n* = 17; *Tbx3* I155S, *n* = 17; *Tbx3* A280S, *n* = 17). Two mice from the I155S group were excluded because of technical issues. The results displayed are an aggregation of 2 independent experiments. (**F**) Quantification of the triglyceride secretion rate of mice from **D**. The results displayed are an aggregation of 2 independent experiments. (**G**) Liver weight (left), body weight (middle), and liver/body weight ratios of WT C57BL/6 mice injected with AAV expressing *GFP*, WT *Tbx3*, or *Tbx3* harboring the I155S or A280S mutations fed a WD for 12 weeks. (**H**) Representative H&E images of mice from **G**. Scale bars: 500 μm, left panels; 100 μm, right panels. Significance of the difference in plasma triglycerides among all groups at 180 minutes after P407 injection in **E** was calculated using a 2-way ANOVA with Tukey’s post hoc test. The statistics displayed in **E** represent significance of each group compared with the *Tbx3-*WT group. Significance of the difference in triglyceride secretion rates and the difference in liver weight, body weight, and liver/body weight ratio comparing each group to *Tbx3* WT in **F** and **G** was done using a 1-way ANOVA with Tukey’s post hoc test. *P < 0.05; **P < 0.01; ***P < 0.001; ****P < 0.0001.

Loss of murine *Tbx3* accelerates VLDL particle export in the presence of MASLD, and sequencing of fatty livers revealed mutations of unknown consequence throughout human *TBX3* (Figure 1C). To characterize the functional impact of these mutations, we generated V5-tagged WT and mutant *Tbx3* expression constructs. Based on the high ΔΔG from AlphaFold predictive models (Figure 1E), we hypothesized that some of the mutations destabilize TBX3. To test whether these mutations alter protein stability, we transfected WT H2.35 cells with WT or point mutant constructs. Twenty-four hours after transfection, we detected less V5-TBX3 harboring the I155S point mutation, whereas the protein levels of TBX3 G35C, F40L, and A280S were unchanged (Figure 7A). Next, we asked whether any of these mutations alter the subcellular localization of TBX3 using immunofluorescence on H2.35 cells. While *TBX3* WT and G35C and F40L mutants localized mostly to the nucleus, I155S and A280S were found in the nucleus and cytoplasm, indicating loss of nuclear localization (Figure 7B). These mutations are within the T-box DNA binding domain, and the nuclear localization signal is found adjacent to this domain. We hypothesized that these DNA binding domain mutations affected nuclear localization and transcriptional functionality. To test whether these mutations prevented TBX3-mediated suppression of secretion, we cotransfected WT H2.35 cells with a Gaussia luciferase and different *Tbx3* constructs. The DNA binding domain mutants, in particular I155S, failed to suppress secretion of Gaussia luciferase to the same extent as WT TBX3, whereas the mutations found upstream of the DNA-binding domain were similar to the WT (Figure 7C).

To assess mutations in vivo, we injected WT mice with AAV-TBG-TBX3 containing the I155S or A280S mutations, and performed in vivo poloxamer-407 secretion assays after 2 weeks of WD feeding. Consistent with the in vitro data, 1 week after AAV injection, we observed less V5-positive staining in livers injected with TBX3 I155S (Figure 7D). While WT TBX3 suppressed VLDL secretion, I155S and A280S point mutants showed a complete inability to do so (Figure 7, E and F). Finally, after 12 weeks of WD feeding, I155S and A280S had decreased lipid accumulation relative to WT TBX3 and were comparable to the GFP control (Figure 7, G and H, and Supplemental Figure 6I). This showed that *TBX3* somatic mutations identified in human patients are predominantly loss-of-function, and likely promote MASLD-driven clonal expansion through accelerated VLDL particle secretion.

## Discussion

Human tissues contain diverse somatic mutations, and aging and disease can accelerate somatic evolution. Clonal evolution is driven by not only the somatic mutations themselves, but the cellular environment within which the mutations arise. In order to capitalize on the genetic information gained from somatic genetics, it will be essential to distinguish between passenger and driver mutations, and to identify the specific mechanisms that underlie their expansion. To identify gene perturbations that drive expansion during MASLD, we used the MOSAICS screening platform, which identified loss-of-function *Tbx3* mutations that expand in MASLD livers.

The implications of somatic mutations for disease progression and organismal health are unclear. One possibility is that these mutations are precursors to transformation and ultimately tumorigenesis. However, mutations could also be beneficial or adaptive, causing increased regeneration within damaged tissues. In the esophagus, clonally expanding epithelial cells restrict tumorigenesis by outcompeting emerging cancer cells (29, 30). In the liver, several mutations found in cirrhotic livers are not commonly found in hepatocellular carcinoma. Importantly, some of these mutations induce liver regeneration without promoting cancer (31, 32). These results call into question the idea that somatic mutations are predominantly harmful. Instead, they raise the possibility that some somatic mutations, even at low frequencies, could be beneficial to the health of an organism in some contexts. Here, we show that mutations in *TBX3* provide a selective advantage in MASLD at the cellular level by accelerating the rate of VLDL-TG secretion. We further provide evidence that secreting lipids, rather than storing them, is a general mechanism that drives expansion of hepatocyte clones during MASLD development, including *TBX3* mutant clones. Notably, *TBX3* mutations increase the rate of VLDL particle secretion, resulting in an elevated VLDL particle number. This differs from other genes implicated in VLDL particle formation and MASLD, such as *TM6SF2*, which regulates the number of triglycerides per VLDL particle without influencing the number of VLDL particles secreted (33).

These results suggest that TBX3 assists in maintaining cholesterol homeostasis, which plays a role in its suppression of VLDL secretion and may also be important for its role in embryogenesis. In rats, pharmacologic inhibition of cholesterol synthesis causes developmental abnormalities (34). In humans, inborn errors of cholesterol synthesis induce different malformation syndromes (35), many of which present clinically with underdeveloped limbs, mirroring features of ulnar mammary syndrome caused by germline *TBX3* mutations. TBX3 could play a unique role in maintaining proper cholesterol levels specifically early in embryogenesis, when much of the fetal cholesterol comes exogenously from the mother (36).

While inhibition of TBX3 may be useful in protecting from MASLD, it likely is not a viable treatment option owing to potential side effects. Despite being protected from MASLD, *Tbx3*-KO mice faced other health challenges, such as increased systemic insulin resistance. High-dose triglyceride infusion is a common method of inducing acute insulin resistance in mice and humans (37), so it is possible that chronic hyperlipidemia resulting from accelerated VLDL secretion exacerbated the insulin resistance phenotype caused by the WD. Furthermore, elevated levels of VLDL and LDL particles are known to cause atherosclerosis and cardiovascular disease (38). This study is important because it shows that somatic mutations can have potent beneficial effects at the clone, tissue, and organ levels, but also a detrimental effect on the individual. This shows that potential inter-organ trade-offs of somatic mutations must be quantified and analyzed in the in vivo context before therapeutic conclusions can be made.

## Methods

### Sex as a biological variable.

For the mouse MASLD phenotype, both male and female mice were used because sex was considered as a biological variable. For the remaining experiments, male mice were used because in general they exhibit more rapid and severe fatty liver disease development.

### Sequencing of human MASLD patients and generation of predictive models.

Sequencing was performed as described by Ng et al. (10). To predict the impact of *TBX3* mutations on protein stability and function, *TBX3* mutations were mapped onto the full-length TBX3 structure predicted by AlphaFold (https://alphafold.ebi.ac.uk/) or the TBX3: DNA complex x-ray crystal structure (Protein Data Bank [PDB] ID: 1H6F) (17) using PyMOL (https://www.pymol.org/). The impact of T-box mutations on TBX3 stability was predicted by importing of the TBX3: DNA x-ray crystal structure coordinates into the DDMut online server (18) and querying of individual mutations of interest.

### Mouse models of fatty liver disease.

We received *Tbx3^fl/fl^* mice on a mixed FVB/129/B6 strain background from Anne Moon’s laboratory at the University of Utah and backcrossed them for 2 generations onto C57BL/6. For the *Tbx3*/*Hdlbp* double-knockout experiment, *Rosa-Rtta TRE-Cas9* mice on a C57BL/6 background (The Jackson Laboratory, 029415) were used. For all experiments that included *TBX3* overexpression (Figures 3 and 7), WT C57BL/6 mice were originally purchased from The Jackson Laboratory. We used the WD fatty liver disease model described previously (32). The diet contains solid food high in fat, cholesterol, and sucrose (Teklad Diets, TD.120528) and a high-sugar water with 23.1 g/L d-fructose (Sigma-Aldrich, G8270) and 18.9 g/L d-glucose (Sigma-Aldrich, F0127). Methionine- and choline-deficient HFD was used as an additional fatty liver disease model (Research Diets, A06071302) (25). Six- to eight-week old *Tbx3^fl/fl^* mice were injected with 5 × 10^10^ genome copies (GC) of AAV-TBG-Cre or AAV-TBG-GFP, sufficient for whole liver coverage. AAV-TBG-GFP control mice were generated from littermates and cohoused with mice injected with AAV-TBG-Cre for each study. One week after AAV injection, the mice were switched to the indicated diets for the length of the study. At the endpoint of the studies, blood was collected from the submandibular vein for ALT, AST, cholesterol, triglyceride, and non-esterified fatty acids analysis, and liver tissue was harvested and processed. For the NC, *Tbx3^fl/fl^* mice were injected with AAV-TBG-Cre or AAV-TBG-GFP and maintained on NC for the duration of the study. To generate *Tbx3* overexpression AAV vector, we cloned WT mouse *Tbx3* with a V5 tag into an AAV backbone driven by a TBG promoter (Addgene 105536). AAV expressing *Tbx3* or *GFP* (3 × 10^10^ GC) were injected into WT C57BL/6 mice. One week after injection, mice were started on WD or maintained on NC for the remainder of the study. For the in vivo point mutant secretion and MASLD studies, 5 × 10^10^ GC were injected into WT C57BL/6 mice.

### Mouse somatic Tbx3 mutations.

We used the Site-Directed Mutagenesis Kit (New England Biolabs, E0552S) to generate lentiviral expression plasmids expressing a V5-tagged *Tbx3* containing somatic mutations. For TBX3 localization studies, WT H2.35 cells were sparsely seeded onto plates with a glass bottom (Thermo Fisher Scientific, 150680). The next day, cells were transfected with one of the lentiviral expression plasmids containing WT or point mutant *Tbx3*. Twenty-four hours after transfection, cells were washed with PBS and immunofluorescence was performed as described below. For Gaussia luciferase secretion assay, WT H2.35 cells were seeded into a 12-well plate. The next day, cells were cotransfected with the Gaussia luciferase expression plasmid and WT, or point mutant *Tbx3*. Twenty-four hours after transfection, secretion was measured as described below. To express point mutant *Tbx3* in mouse livers, we cloned point mutant *Tbx3* cDNAs into the same AAV backbone as before. Mice were injected with AAV and started on WD one week later. After 2 weeks, mice were subjected to the in vivo VLDL-TG secretion assay described below. After 12 weeks on WD, tissue was harvested and analyzed.

### Tbx3/Hdlbp double-knockout livers.

sgRNAs targeting *Tbx3* and *Hdlbp* along with *LacZ* and a non-targeting control were cloned into the MOSAICS v10 vector. AAV was generated and purified as described below. At 7 weeks old, *Rosa-Rtta TRE-Cas9* mice were given doxycycline water (2 g/L) for 2 weeks. After 1 week on doxycycline, mice were injected with 1 × 10^11^ GC of MOSAICS v10 vectors. The mice were maintained on doxycycline water for an additional week after AAV injection. One week after AAV injection, doxycycline water was removed, and the mice were given WD for 2 weeks before an in vivo VLDL-TG secretion assay described below.

### AAV generation and purification.

AAV-Pro 293T cells (Takara 632273) were seeded at a density of 2 × 10^7^ cells per 15 cm plate 1 day before transfection. For transfecting one 15 cm plate, 10 μg of AAV-TBG plasmid, 10 μg of pAAV2/8 (Addgene 112864), and 20 μg of pAdDeltaF6 (Addgene) were mixed with 500 μL of Opti-MEM (Thermo Fisher Scientific). In another tube, polyethyleneimine (PEI) was mixed with 500 μL Opti-MEM. The solutions from both tubes were mixed and incubated at room temperature for 30 minutes before being added to the plates. The next day, cell culture medium was replaced with low-glucose DMEM supplemented with 1% FBS and 1× penicillin/streptomycin. Forty-eight hours later, cells were scraped off the plate and collected by centrifugation, and the pellets were frozen at –80°C overnight. The next day, cells were thawed on ice and lysed in 2 mL per 15 cm plate of lysis buffer (200 mM NaCl in PBS supplemented with 0.5% CHAPS powder [wt/vol]). The lysates were rocked at 4°C for 15 minutes, centrifuged, and filtered with 0.45 μm filters. During centrifuging, the gravity column for AAV purification was set up. In a Poly-Prep chromatography column (Bio-Rad, 7311550), 0.8 mL of POROS CaptureSelect AAV8 Affinity Resin (Life Technologies, A30790) was added to the bottom of the column and washed with 5 mL of wash buffer (500 mM NaCl in PBS). After washing, the filtered cell lysate containing AAV was added to the resin without disturbing it and allowed to run through. Each column was then washed again with 8 mL of wash buffer before being eluted directly into 1 M Tris-HCl (pH 8) with the elution buffer (500 mM NaCl, 100 mM glycine [pH 2.5]). The eluate was transferred to a 100K Amicon centrifugal filter unit (Millipore, UFC910024) and concentrated by centrifugation. The concentrated AAV was diluted with 3 mL of AAV dialysis buffer (212 mM NaCl and 5% sorbitol [wt/vol]) and concentrated again. After concentration, the AAV was again diluted with 3 mL of AAV dialysis buffer and concentrated a third time. The final concentrated AAV was aliquoted and stored at –80°C until it was used. AAV titer was determined using the AAVpro titration kit (for Real Time PCR) version 2 (Takara 6233).

### Glucose tolerance test and fasted plasma insulin concentration.

The glucose tolerance test (GTT) assay was performed 22 weeks after WD feeding was initiated. Peripheral blood for fasted insulin was collected 24 weeks after WD feeding was initiated. Mice were fasted overnight for 16 hours before the GTT test. A small opening was made with a blade on the lateral tail vein for blood glucose testing. Fasted glucose levels were measured and used as time point 0. Ten percent glucose was injected i.p. at 10 μL per gram of body weight. The dosage was 1 g/kg body weight. Plasma glucose levels at 30, 60, 90, and 120 minutes were measured with a LifeScan OneTouch glucometer. For fasting insulin concentration, mice were fasted for 5 hours before testing. After that, blood was collected from the lateral tail vein and collected into heparinized capillaries (Drummond Scientific 1-000-7500-HC). Peripheral blood was centrifuged, and plasma was transferred to a fresh tube and stored at –80°C. Insulin concentrations were measured using the Ultra Sensitive Mouse Insulin ELISA kit (Crystal Chem 90080).

### Lipoprotein fractionation.

Fresh plasma was collected and pooled from 4–5 mice from each group and fractionated by the University of Texas (UT) Southwestern Medical Center Metabolic Phenotyping Core. Samples were separated using a Superose 6 10/300 GL gel filtration column, and triglycerides were measured from each fraction.

### In vivo VLDL-TG secretion assay and ApoB concentration.

In vivo VLDL-TG secretion assay was performed on mice after 2 weeks on WD. Mice were fasted overnight before the secretion assay. Peripheral blood was collected in heparinized tubes from the submandibular vein from fasted mice, which served as time point 0. Poloxamer-407 (Sigma-Aldrich, 16758) was dissolved in saline, filtered, and given i.p. at a dose of 1 g/kg. Ninety minutes and 180 minutes after poloxamer injection, peripheral blood was collected with heparinized capillaries. The blood was centrifuged, and the plasma was transferred to a fresh tube and stored at –80°C until analysis. Triglycerides were measured from the plasma by the UT Southwestern Metabolic Phenotyping Core. We calculated VLDL secretion rates by subtracting the final TG concentration from the fasted triglyceride concentration and dividing by the total time. In *Tbx3-*KO mice, plasma ApoB concentration was measured using a mouse ApoB ELISA kit (Elabscience, E-EL-M3017).

### CUT&RUN and ChIP-Seq library preparation and analysis.

ChIP-Seq DNA was prepared from mouse livers as described previously using a Ty1 antibody (Diagenode, C15200054) (39). DNA libraries were prepared using the NEBNext Ultra II DNA Library Prep Kit for Illumina (New England Biolabs, E7645). For CUT&RUN DNA preparation, low-passage-number cell lines were harvested, and genomic binding sites were isolated according to the manufacturer’s protocol (Epicypher, 14-1048) using a V5 antibody (Abcam, ab15828). Next-generation sequencing libraries were also prepared according to the manufacturer’s protocol (Epicypher, 14-1001). Samples were sequenced with 50-bp paired-end flow cells using an Illumina NextSeq 2000 system at the Children’s Research Institute Sequencing Core.

All FASTQ files were trimmed via Cutadapt (: https://cutadapt.readthedocs.io/en/stable/) and aligned via Bowtie 2 (https://bowtie-bio.sourceforge.net/bowtie2/index.shtml) to the UCSC reference mouse genome. Peaks were called using MACS2 (https://macs3-project.github.io/MACS/index.html). All resulting bigWig files were visualized by the Integrative Genomics Viewer (https://igv.org/). Heatmaps displaying promoter regions within the mouse genome were curated using deepTools (https://deeptools.readthedocs.io/en/latest/).

### In vivo β-oxidation and free fatty acid flux.

[^13^C]potassium palmitate (Cambridge Isotope Laboratories, CLM-3943-PK) was resuspended at a concentration of 50 mM and mixed 1:10 in PBS containing 10% fatty acid–free BSA (Sigma-Aldrich, A8806). Mice were infused at a rate of 1 mg/kg/min for 2 hours, and then tissue was immediately collected. We performed metabolomics, and β-oxidation flux was measured by calculation of the ratio of M+14 myristoylcarnitine to M+16 palmitoylcarnitine in the liver. To calculate free fatty acid uptake, we performed lipidomics and calculated the fractional enrichment of M+16-labeled palmitate in the plasma and liver.

### Histology and immunohistochemistry.

Tissue samples were fixed with 10% formalin and paraffin-embedded. Slides were deparaffinized using serial incubations with xylene, xylene, 100% ethanol, 100% ethanol, 90% ethanol, 80% ethanol, 70% ethanol, 50% ethanol, 30% ethanol, and H_2_O, followed by antigen retrieval using Citra Plus (Biogenex Laboratories, HK0809K). Slides were then blocked with 3% hydrogen peroxide in methanol, washed with PBS followed by 10% goat serum–PBST, and then incubated with primary antibody diluted in 10% goat serum–PBST overnight at 4°C. After washing with 1× PBST 3 times for 5 minutes each, slides were incubated with biotin-labeled secondary antibody and detected using the VECTASTAIN Elite ABC HRP Kit (Vector Laboratories, PK6100) and DAB with HRP Substrate Kit (Vector Laboratories, SK4100). Primary antibody used for immunohistochemistry was V5 (1:100) (Cell Signaling Technology, 13202). For Sirius red staining, slides were deparaffinized as described above, and Sirius red staining was performed using a Picro Sirius Red staining kit (Abcam, ab150681) according to the manufacturer’s protocol.

### Immunofluorescence.

Cells were prepared as described above. Twenty-four hours after transfection, cells were washed with PBS and fixed with 4% paraformaldehyde (Thermo Fisher Scientific, AAJ19943K2) for 10 minutes. Cells were washed 3 times in PBS for 5 minutes each time, and permeabilized with 0.5% Triton 100 for 10 minutes. Again, cells were washed 3 times in PBS for 5 minutes each time and blocked in 5% BSA in PBS for 1 hour. After blocking, cells were incubated at 4°C with primary antibody diluted in blocking buffer overnight. The next day, cells were washed 3 times with 0.05% Triton X-100 in PBS for 5 minutes each time, and secondary antibody diluted in blocking buffer was added to the cells for 1 hour at room temperature. Cells were washed 3 times with 0.05% Triton and were stained with Hoechst 33342 for 15 minutes, washed with deionized water, and imaged immediately. Pictures were taken with a spinning disk confocal microscope from the UT Southwestern Live Cell Imaging Facility. Images were analyzed in ImageJ (NIH), but visualized using NIS elements image software (URL: https://www.microscope.healthcare.nikon.com/products/software/nis-elements/software-resources). Antibodies used for immunofluorescence were anti-V5 (1:1,000) (Cell Signaling Technology, 13202) and anti-rabbit IgG Alexa Fluor 647 (1:500) (Thermo Fisher Scientific, A-21244).

### RNA extraction and qPCR.

Total RNA was purified using Trizol, followed by chloroform extraction, isopropanol precipitation, and ethanol washing. Precipitated pellets were allowed to dry before being reconstituted in nuclease-free water. RNA concentration was measured using a NanoDrop spectrophotometer (Thermo Fisher Scientific) and diluted to reach a final concentration of 100 ng/μL. cDNA was prepared in a thermocycler from 1 μg of RNA using iScript (Bio-Rad, 1708891) with the following conditions: 25°C for 5 minutes, 42°C for 45 minutes, 95°C for 1 minute. cDNA was diluted 10-fold, and qPCR was done using SYBR Green (Bio-Rad, 1725125).

### Cell culture studies.

HEK293T cells (ATCC, CRL-3216) were cultured in complete DMEM supplemented with 10% FBS and 1× penicillin/streptomycin. H2.35 cells were cultured with complete DMEM supplemented with 4% FBS, 200 nM dexamethasone, and 1× penicillin/streptomycin. H2.35 mouse hepatocyte cells (ATCC, CRL-1995) were infected with lentivirus expressing a V5-GFP, V5-TBX3, Cas9/*Gal4*, or Cas9/mouse *Tbx3* sgRNA. Cells were selected with 10 μg/mL blasticidin for at least 6 days before experiments. *Tbx3* deletion and overexpression were validated by Western blot.

To measure the Gaussia luciferase secretion in *Tbx3*-KO and -overexpressing cell lines, cells from each genotype were seeded into a 6-well plate and allowed to adhere. The next day, cells were transfected with a Gaussia luciferase expression plasmid (Thermo Fisher Scientific, 16147) along with a fluorescently tagged plasmid to track transfection efficiency. Twenty-four hours after transfection, a sample of cell culture medium was collected, and luciferase activity was measured using a Pierce Gaussia Luciferase Glow Assay Kit (Thermo Fisher Scientific, 16160). For measurement of Gaussia luciferase secretion in HEK293T cells, WT HEK293T cells were seeded into a 12-well plate. The following day, they were cotransfected with a Gaussia luciferase expression plasmid and a siRNA targeting *TBX3* or siCTRL. Forty-eight hours after transfection, Gaussia luciferase activity in the medium was measured as before.

### Protein extraction and Western blotting.

Protein was extracted from mouse liver tissue using T-PER Tissue Protein Extraction Reagent (Thermo Fisher Scientific, 78510) containing freshly added 1× protease inhibitor (ApexBio, K10070) and 1× phosphatase inhibitor (Thermo Fisher Scientific, 501905547). H2.35 cell lines were lysed with 1× RIPA buffer (Life Technologies, 89900) with 1× protease inhibitor and 1× phosphatase inhibitor cocktail. Samples were treated with 6× Laemmli SDS Sample Buffer R (Boston BioProducts, BP-111R-25ml) and heated at 95°C for 5 minutes. Approximately 20 μg of protein per sample (liver tissue) or 5 μg (H2.35 cells) was separated using a Bio-Rad 4%–20% gradient Tris-Glycine SDS Mini gel system and analyzed using the following antibodies: anti-TBX3 (1:10,000) (Bethyl Laboratories, A303-098A), anti-HDLBP (1:5,000) (Abcam, ab109324), anti-V5 (1:1,000) (Cell Signaling Technology, 13202), anti–β-actin (1:5,000) (Cell Signaling Technology, 4970), and anti–cleaved PARP (1:1,000) (Cell Signaling Technology, 94885).

### Statistics.

Variation in all panels is indicated using standard error of the mean presented as mean ± SEM. Two-tailed, unpaired Student’s *t* tests were used to test the significance of differences between 2 groups unless otherwise indicated in the text or figure legends. Statistical significance is displayed as not significant (*P* ≥ 0.05) or the *P* value itself, *P < 0.05, **P < 0.01, ***P < 0.001, ****P < 0.0001, unless specified otherwise.

### Study approval.

All mice were handled in accordance with the guidelines of the Institutional Animal Care and Use Committee at the University of Texas Southwestern Medical Center under approved protocol APN 2015-101118.

### Data availability.

The next-generation sequencing data generated were uploaded to the NCBI’s Gene Expression Omnibus (GEO) repository database (accession number GSE300981). All other data are available within the article, supplemental information, and Supporting Data Values file.

## Author contributions

GM designed research studies, conducted experiments, acquired and analyzed data, and wrote the manuscript. GQ and BL analyzed sequencing data. MZ conducted experiments and provided reagents. ZW and XW conducted experiments. MHH analyzed data. TM, LZ, and WG performed metabolomics and lipidomics, and assisted with data analysis. PG performed NAS scoring. NB, PC, and MH provided human patient sequencing data. GL conducted in silico modeling experiments and ddMut calculations. HZ acquired funding, conceived of the project, designed research studies, analyzed data, and wrote the manuscript.

## Supplementary Material

Supplemental data

Unedited blot and gel images

Supplemental table 1

Supporting data values

## Version 1 07/10/2025

In-Press Preview

## Version 2 09/16/2025

Electronic publication

## Version 3 09/19/2025

Addition of missing funding source
