# Supplementary material for: Somatic mutations in TBX3 promote hepatic clonal expansion by accelerating VLDL secretion
Source: J Clin Invest. 2025 Jul 10;135(18):e191855. doi: 10.1172/JCI191855 (PMC12435840; doi:10.1172/JCI191855)
Supplement: Supplemental data [file jci-135-191855-s008.pdf]

Somatic mutations in *TBX3* promote hepatic clonal expansion by accelerating VLDL secretion

Authors:

Gregory Mannino[1], Gabriella Quinn[2], Min Zhu[1], Zixi Wang[1], Xun Wang[1], Boyuan Li[1], Meng-Hsiung Hsieh[1], Thomas Mathews[1], Lauren Zacharias[1], Wen Gu[1], Purva Gopal[3], Natalia Brzozowska[4], Peter Campbell[4,5], Matt Hoare[6], Glen Liszczak[7], Hao Zhu[1]

Affiliations:

[1]Children's Research Institute, Department of Pediatrics and Internal Medicine, Center for Regenerative Science and Medicine, Simmons Comprehensive Cancer Center, Children's Research Institute Mouse Genome Engineering Core, University of Texas Southwestern Medical Center, Dallas, TX 75390, USA.

[2]Department of Immunology, University of Texas Southwestern Medical Center, Dallas, TX 75390, USA.

[3]Department of Pathology, University of Texas Southwestern Medical Center, Dallas, TX 75390, USA.

[4]Cancer Genome Project, Wellcome Sanger Institute, Hinxton, Cambridgeshire CB10 1SA, UK.

[5]Quotient Therapeutics, Little Chesterford, Saffron Walden CB10 1XL, UK.

[6]University of Cambridge Department of Medicine, Cambridge Biomedical Campus, Cambridge, CB2 0QQ, UK and University of Cambridge Early Cancer Institute, Hutchison Research Centre, Cambridge Biomedical Campus, Cambridge, CB2 0XZ, UK.

[7]Department of Biochemistry, University of Texas Southwestern Medical Center, Dallas, TX 75390, USA.

*Corresponding author: Hao Zhu, 5901 Forest park Road, NL10.120C, Dallas, Texas 75390, USA. Phone: 214.648.2850. Email: Hao.Zhu@utsouthwestern.edu

SUPPLEMENTAL FIGURES

Supplemental Figure 1

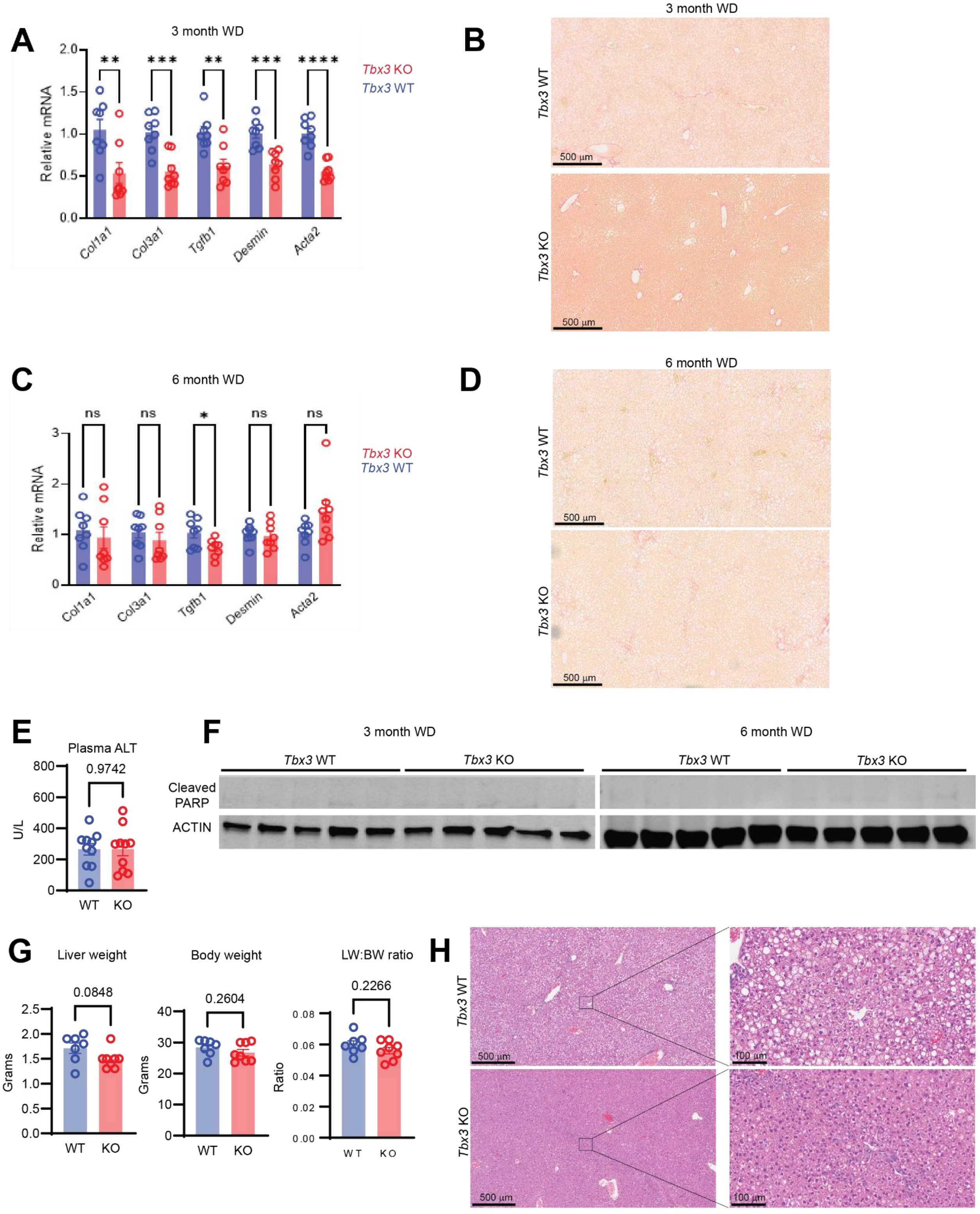

Supplemental Figure 1. Loss of *Tbx3* protects against WD induced MASLD.

- **A.** qPCR for fibrosis markers in *Tbx3* WT or KO mice fed WD for 3 months.
- **B.** Representative Sirius Red staining in mice from A.
- **C.** qPCR for fibrosis genes in *Tbx3* WT or KO mice fed WD for 6 months.
- **D.** Representative Sirius Red staining in mice from C.
- **E.** Plasma ALT in mice from C.
- **F.** Western blot for cleaved PARP in *Tbx3* KO or WT mice fed WD for 3 (left) and 6 (right) months.
- **G.** Liver weight (left), body weight (middle) and liver:body weight ratio from female *Tbx3* KO or WT mice fed WD for 3 months.
- **H.** Representative H&E image from mice in G.

Supplemental Figure 2

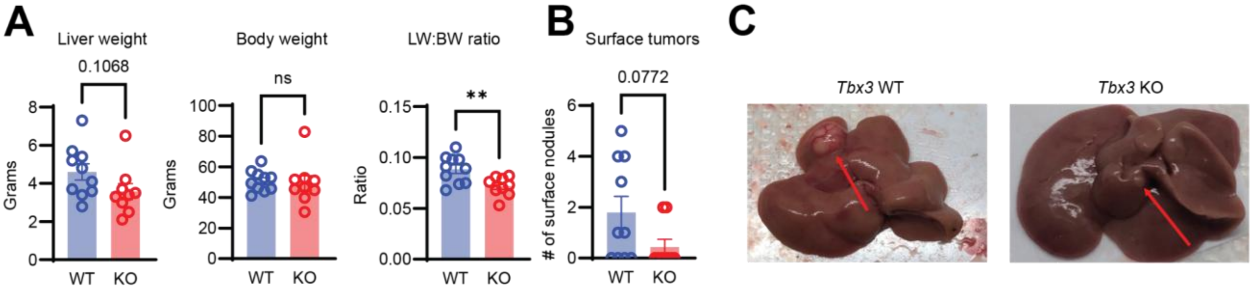

Supplemental Figure 2. Loss of *Tbx3* protects against MASLD induced liver tumors.

- **A.** Liver weight (left), body weight (middle), and LW:BW ratio (right) of *Tbx3* WT or KO mice fed a WD for 48 weeks.
- **B.** Surface tumors from mice in A.
- **C.** Example of large surface tumor from mice in A.

Supplemental Figure 3

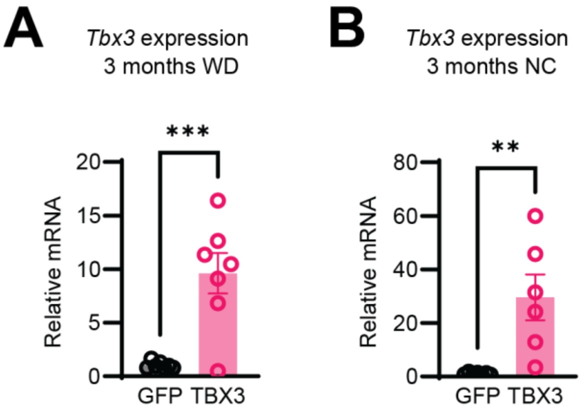

Supplemental Figure 3. MASLD exerts negative selective pressures on *Tbx3* expressing hepatocytes.

A. *Tbx3* expression in TBX3-V5 or GFP-V5 injected mice after 3 months of WD feeding.
B. *Tbx3* expression in Tbx3-V5 or GFP-V5 injected mice after 3 months of NC feeding.

Supplemental Figure 4

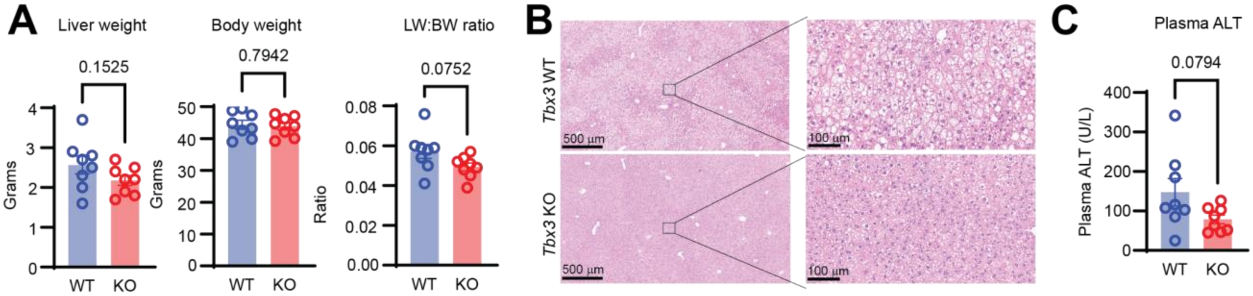

Supplemental Figure 4. *Tbx3* deletion is protective against MASLD associated with aging.

- **A.** Liver weight (left), body weight (middle), and liver:body weight ratio (right) of *Tbx3* WT or KO mice fed NC diet for 6 months.
- **B.** Representative H&E image from mice in A.
- **C.** Plasma ALT from mice in A.

Supplemental Figure 5

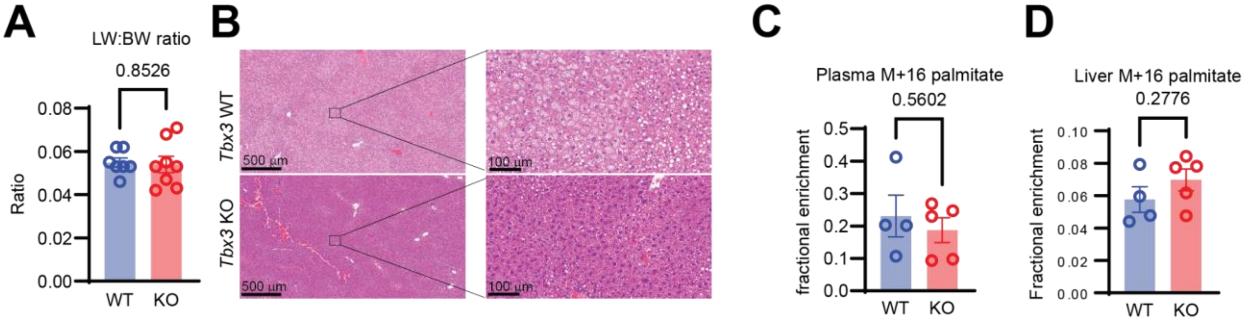

Supplemental Figure 5. *Tbx3* does not alter de novo lipogenesis or free fatty acid uptake.

- **A.** LW:BW ratio of *Tbx3* KO or WT mice fed a WD for 4 weeks.
- **B.** Representative H&E images from mice in A.
- **C.** Fractional enrichment of M+16 palmitate in the plasma of *Tbx3* KO or WT mice infused with ^{13}C potassium palmitate after 2 weeks of WD feeding.
- **D.** Fractional enrichment of M+16 palmitate in the liver from mice in C.

Supplemental Figure 6

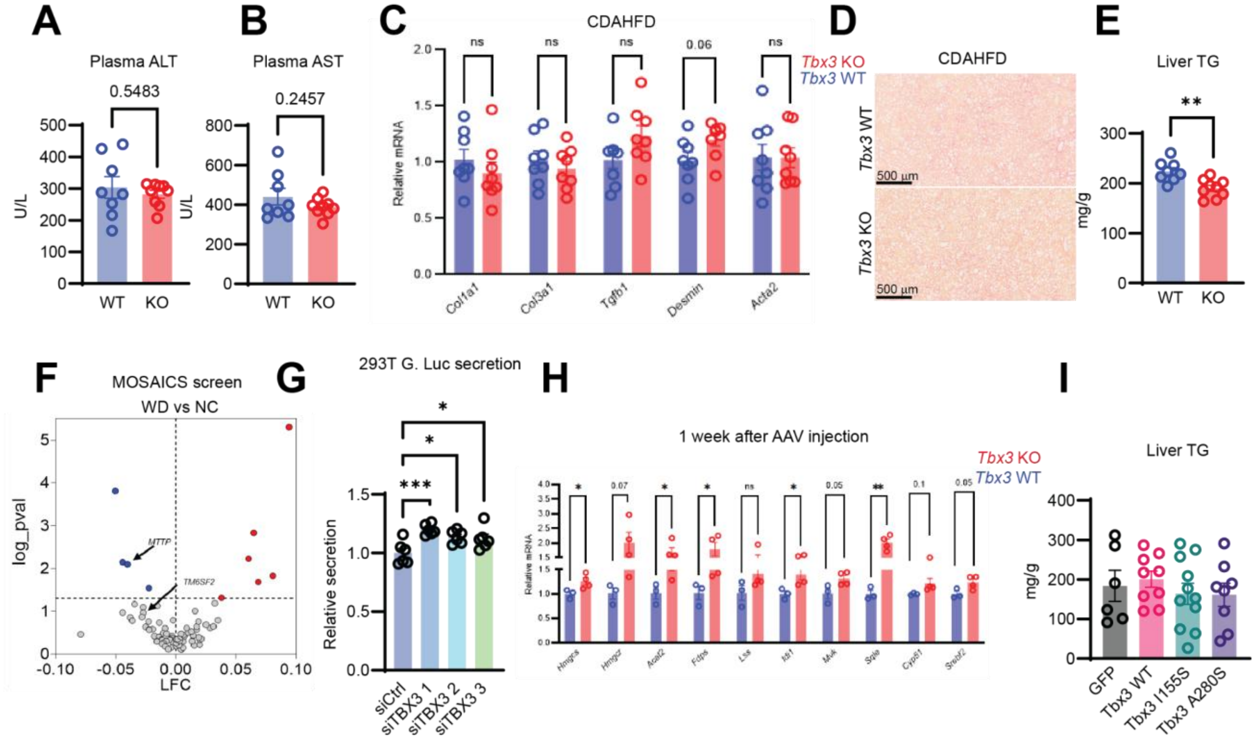

Supplemental Figure 6. Loss of Tbx3 protects from MASLD by increasing VLDL secretion.

A. Plasma ALT in *Tbx3* KO or WT mice fed a CDAHFD for 3 months.
B. Plasma AST from mice in A.
C. qPCR for fibrosis marker genes from mice in A.
D. Representative sirius red staining from mice in A.
E. Liver triglyceride measurements from mice in A.
F. Volcano plot of MOSAICS screen (11) showing depletion of *Mttp* and *Tm6sf2* KO clones in WD vs. NC fed mice.
G. Relative secretion of Gaussia luciferase after *TBX3* knockdown in HEK293T cells.
H. qPCR of cholesterol biosynthesis genes in Tbx3 KO or WT mice 1 week after AAV injection.
I. Liver triglyceride measurements from mice overexpressing GFP-V5, WT TBX3-V5, TBX3-V5 containing a point mutation after 12 weeks of WD feeding.

Relative secretion in G was calculated using a One-way ANOVA corrected for multiple comparisons.

Bibliography

1. Li R, et al. A body map of somatic mutagenesis in morphologically normal human tissues. *Nature*. 2021;597(7876):398–403.

2. Yizhak K, et al. RNA sequence analysis reveals macroscopic somatic clonal expansion across normal tissues. *Science*. 2019;364(6444). https://doi.org/10.1126/science.aaw0726.

3. Kakiuchi N, Ogawa S. Clonal expansion in non-cancer tissues. *Nat Rev Cancer*. 2021;21(4):239–256.

4. Martincorena I, et al. Somatic mutant clones colonize the human esophagus with age. *Science*. 2018;362(6417):911–917.

5. Yoshida K, et al. Tobacco smoking and somatic mutations in human bronchial epithelium. *Nature*. 2020;578(7794):266–272.

6. Olafsson S, et al. Somatic Evolution in Non-neoplastic IBD-Affected Colon. *Cell*. 2020;182(3):672-684.e11.

7. Kakiuchi N, et al. Frequent mutations that converge on the NFKBIZ pathway in ulcerative colitis. *Nature*. 2020;577(7789):260–265.

8. Nanki K, et al. Somatic inflammatory gene mutations in human ulcerative colitis epithelium. *Nature*. 2020;577(7789):254–259.

9. Brunner SF, et al. Somatic mutations and clonal dynamics in healthy and cirrhotic human liver. *Nature*. 2019;574(7779):538–542.

10. Ng SWK, et al. Convergent somatic mutations in metabolism genes in chronic liver disease. *Nature*. 2021;598(7881):473–478.

11. Wang Z, et al. Positive selection of somatically mutated clones identifies adaptive pathways

in metabolic liver disease. *Cell*. 2023;186(9):1968-1984.e20.

12. Khan SF, et al. The roles and regulation of TBX3 in development and disease. *Gene*. 2020;726:144223.

13. Davenport TG, et al. Mammary gland, limb and yolk sac defects in mice lacking Tbx3, the gene mutated in human ulnar mammary syndrome. *Development*. 2003;130(10):2263–2273.

14. Frank DU, et al. Lethal arrhythmias in Tbx3-deficient mice reveal extreme dosage sensitivity of cardiac conduction system function and homeostasis. *Proc Natl Acad Sci USA*. 2012;109(3):E154-63.

15. Bamshad M, et al. The spectrum of mutations in TBX3: Genotype/Phenotype relationship in ulnar-mammary syndrome. *Am J Hum Genet*. 1999;64(6):1550–1562.

16. Jin Y, et al. Wnt signaling regulates hepatocyte cell division by a transcriptional repressor cascade. *Proc Natl Acad Sci USA*. 2022;119(30):e2203849119.

17. Coll M, et al. Structure of the DNA-bound T-box domain of human TBX3, a transcription factor responsible for ulnar-mammary syndrome. *Structure*. 2002;10(3):343–356.

18. Zhou Y, et al. DDMut: predicting effects of mutations on protein stability using deep learning. *Nucleic Acids Res*. 2023;51(W1):W122–W128.

19. Liang W, et al. Establishment of a general NAFLD scoring system for rodent models and comparison to human liver pathology. *PLoS ONE*. 2014;9(12):e115922.

20. Nogueira JP, Cusi K. Role of insulin resistance in the development of nonalcoholic fatty liver disease in people with type 2 diabetes: from bench to patient care. *Diabetes Spectr*. 2024;37(1):20–28.

21. Brown MS, Goldstein JL. Selective versus total insulin resistance: a pathogenic paradox. *Cell Metab*. 2008;7(2):95–96.

22. Ipsen DH, et al. Molecular mechanisms of hepatic lipid accumulation in non-alcoholic fatty liver disease. *Cell Mol Life Sci*. 2018;75(18):3313–3327.

23. Cole LK, et al. Phosphatidylcholine biosynthesis and lipoprotein metabolism. *Biochim Biophys Acta*. 2012;1821(5):754–761.

24. Wang X, et al. Metabolic inflexibility promotes mitochondrial health during liver regeneration. *Science*. 2024;384(6701):eadj4301.

25. Matsumoto M, et al. An improved mouse model that rapidly develops fibrosis in non-alcoholic steatohepatitis. *Int J Exp Pathol*. 2013;94(2):93–103.

26. Badr CE, et al. A highly sensitive assay for monitoring the secretory pathway and ER stress. *PLoS ONE*. 2007;2(6):e571.

27. Zinnall U, et al. HDLBP binds ER-targeted mRNAs by multivalent interactions to promote protein synthesis of transmembrane and secreted proteins. *Nat Commun*. 2022;13(1):2727.

28. Mobin MB, et al. The RNA-binding protein vigilin regulates VLDL secretion through modulation of Apob mRNA translation. *Nat Commun*. 2016;7:12848.

29. Abby E, et al. Notch1 mutations drive clonal expansion in normal esophageal epithelium but impair tumor growth. *Nat Genet*. 2023;55(2):232–245.

30. Colom B, et al. Mutant clones in normal epithelium outcompete and eliminate emerging tumours. *Nature*. 2021;598(7881):510–514.

31. Zhu M, et al. Somatic mutations increase hepatic clonal fitness and regeneration in chronic liver disease. *Cell*. 2019;177(3):608-621.e12.

32. Zhu M, et al. PKD1 mutant clones within cirrhotic livers inhibit steatohepatitis without promoting cancer. *Cell Metab*. 2024;36(8):1711-1725.e8.

33. Smagris E, et al. Inactivation of tm6sf2, a gene defective in fatty liver disease, impairs

lipidation but not secretion of very low density lipoproteins. *J Biol Chem*. 2016;291(20):10659–10676.

34. Roux C, et al. Role of cholesterol in embryonic development. *Am J Clin Nutr*. 2000;71(5 Suppl):1270S–9S.

35. Porter FD, Herman GE. Malformation syndromes caused by disorders of cholesterol synthesis. *J Lipid Res*. 2011;52(1):6–34.

36. Baardman ME, et al. The role of maternal-fetal cholesterol transport in early fetal life: current insights. *Biol Reprod*. 2013;88(1):24.

37. Samuel VT, et al. Lipid-induced insulin resistance: unravelling the mechanism. *Lancet*. 2010;375(9733):2267–2277.

38. Sniderman AD, et al. Apolipoprotein B particles and cardiovascular disease: A narrative review. *JAMA Cardiol*. 2019;4(12):1287–1295.

39. Wang Z, et al. Dual ARID1A/ARID1B loss leads to rapid carcinogenesis and disruptive redistribution of BAF complexes. *Nat Cancer*. 2020;1(9):909–922.
