# Supplementary figures and images for: Somatic mutations in TBX3 promote hepatic clonal expansion by accelerating VLDL secretion
Source: J Clin Invest. 2025 Jul 10;135(18):e191855. doi: 10.1172/JCI191855 (PMC12435840; doi:10.1172/JCI191855)

Supplemental Figure 1F

3 month WD

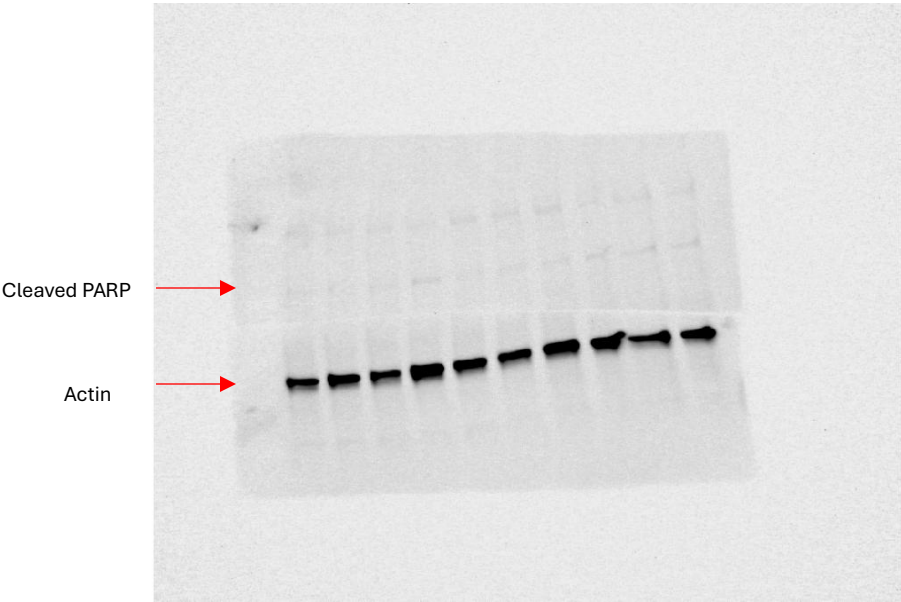

6 month WD

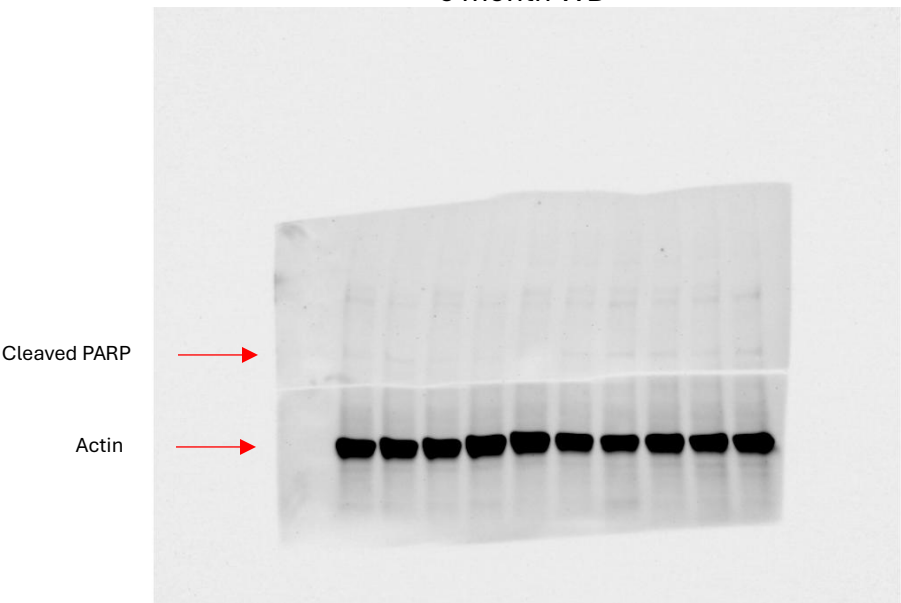

Figure 6A

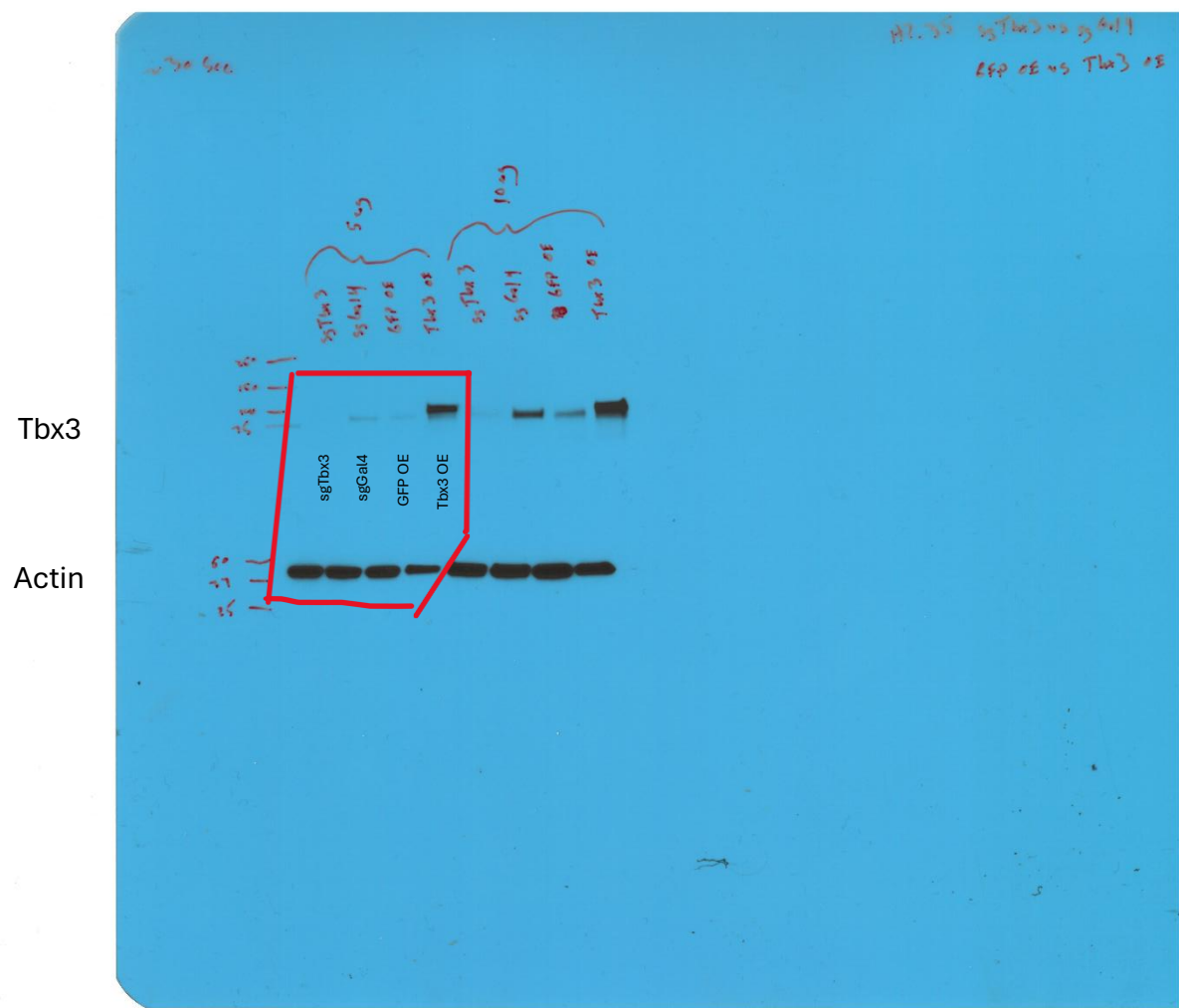

Figure 6H

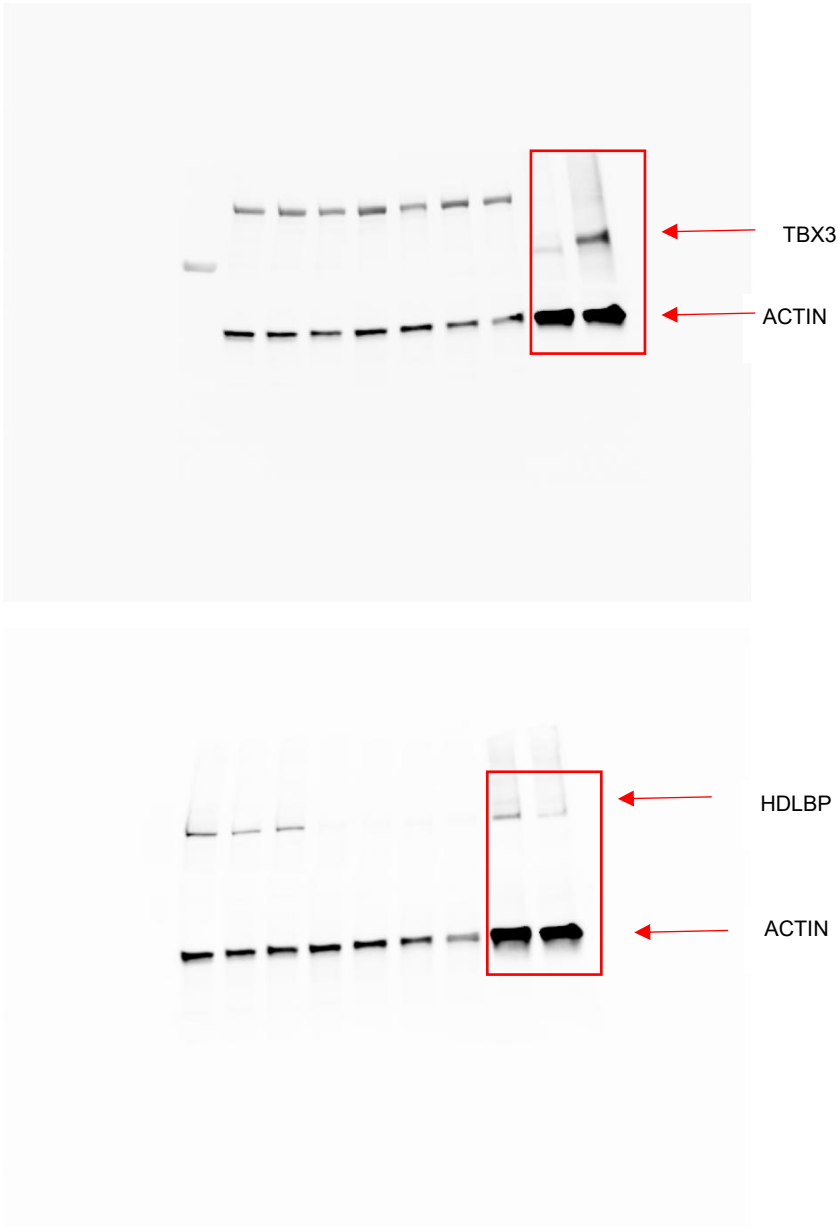

Figure 6J

Tbx3 KO

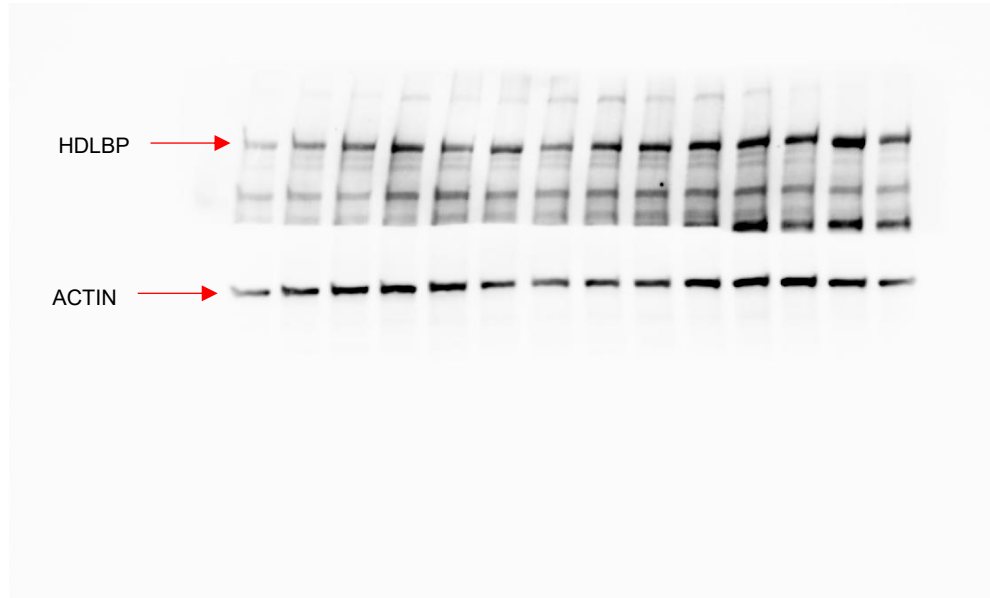

Tbx3 OE

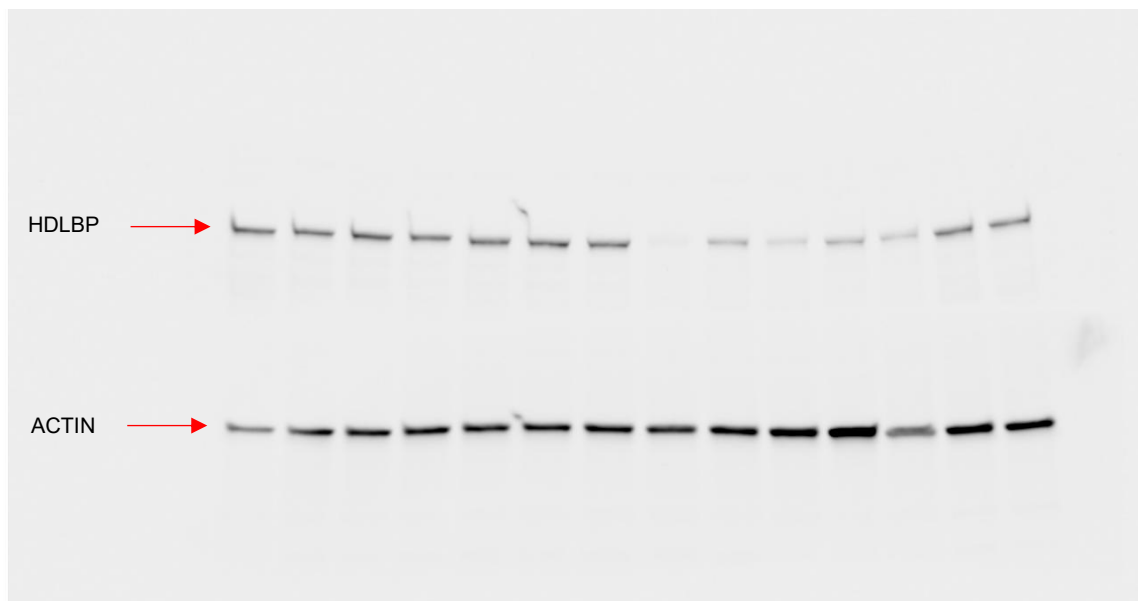

Figure 6K

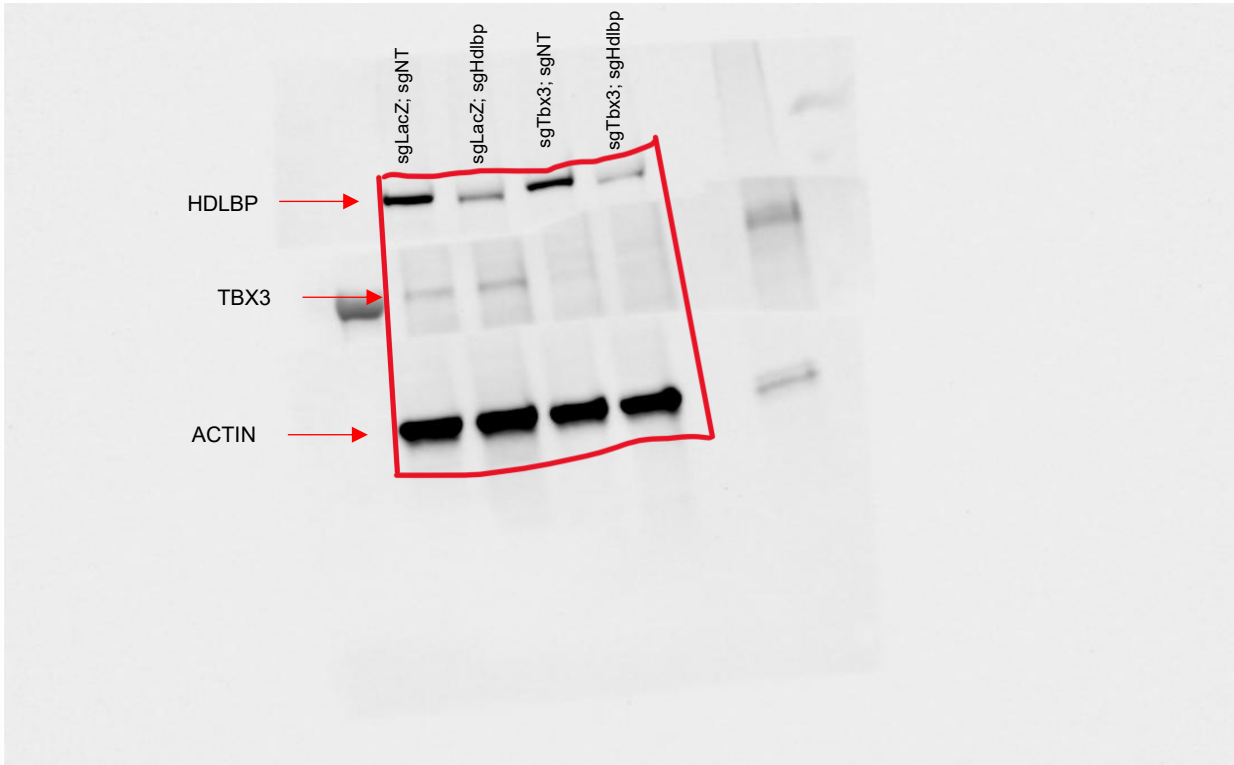

Figure 7A

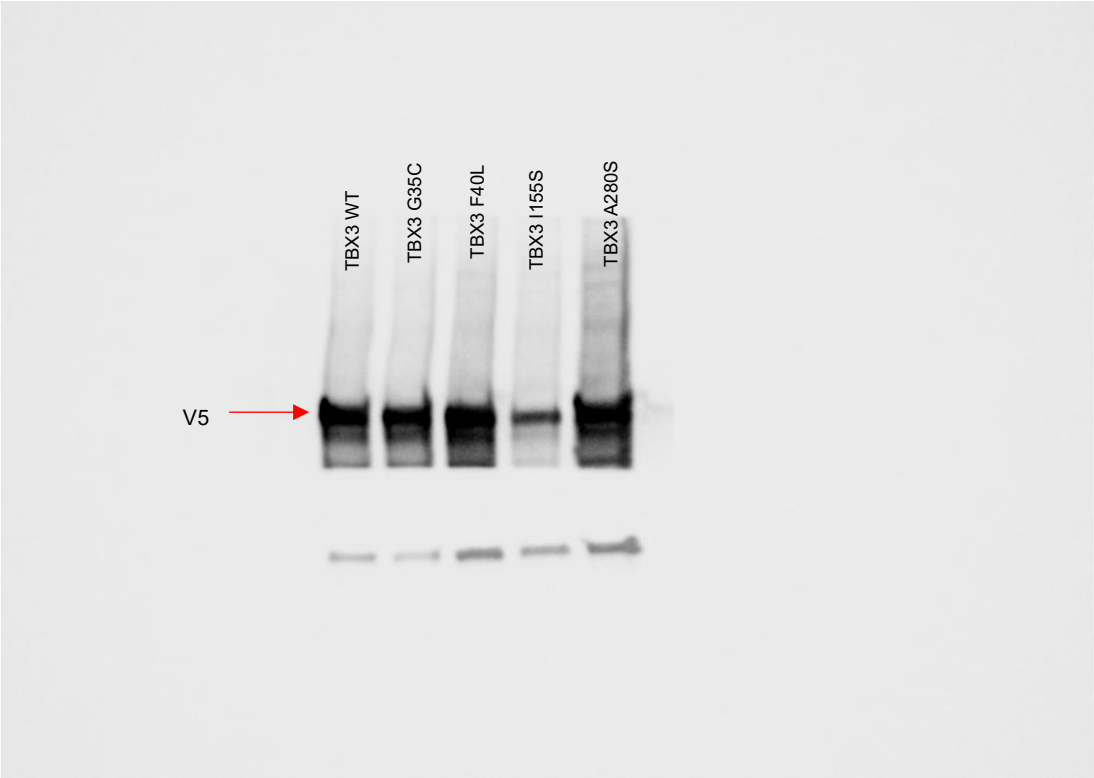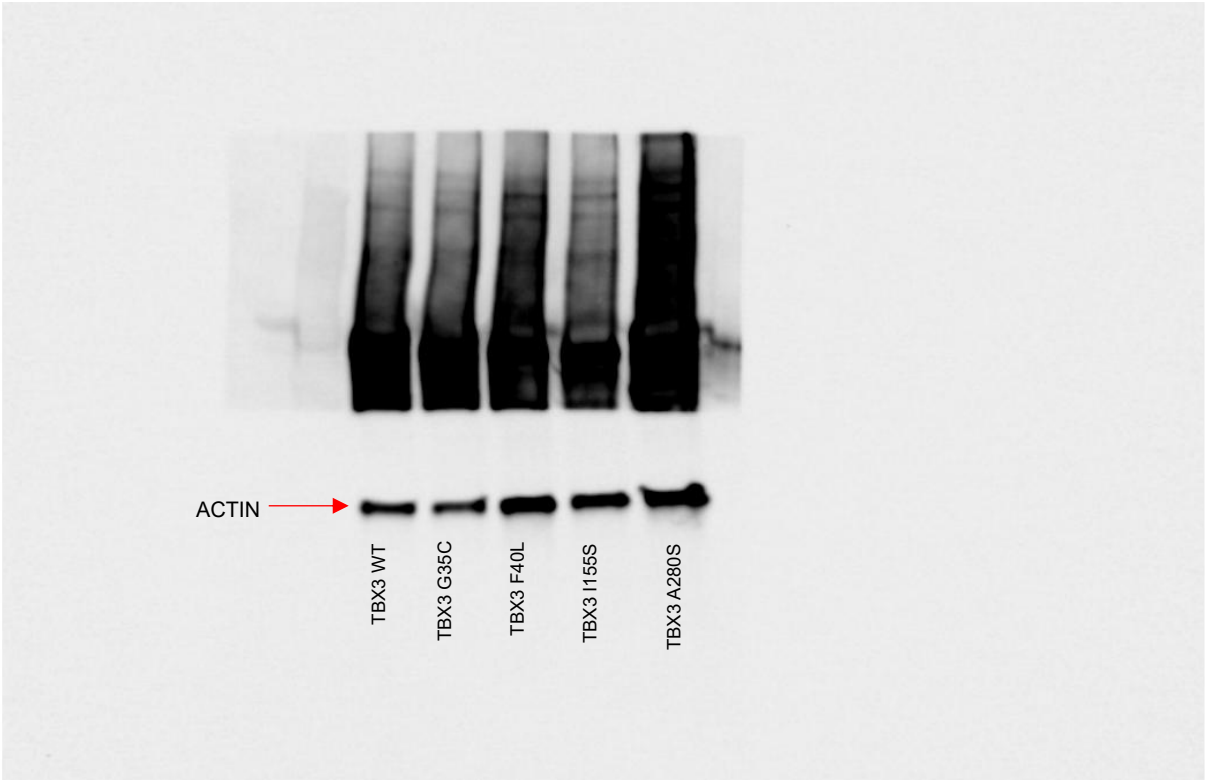

Supplement: Unedited blot and gel images [file jci-135-191855-s009.pdf]
